# Isolation, identification, and pathogenicity comparison of three NADC30-like PRRSV variants with distinct mutational patterns

**DOI:** 10.1128/spectrum.01220-26

**Published:** 2026-10-13

**Authors:** Lei Zhao, Ying Guan, Yang-Ming Dai, Yi-Xin Yan, Shu-Ying He, Tong Xu, Li-Na Shao, Wu Xin, Si-Yuan Lai, Zhi-Wen Xu, Ling Zhu

**Affiliations:** 1 College of Veterinary Medicine, Sichuan Agricultural University 12529, Chengdu, China; 2 Key Laboratory of Animal Diseases and Human Health of Sichuan Province, Chengdu, China; The University of Texas Medical Branch at Galveston, Galveston, Texas, USA

**Keywords:** PRRSV, amino acid deletion in GP5, pathogenicity, phylogenetic analysis, recombination analysis

## Abstract

Porcine reproductive and respiratory syndrome virus (PRRSV) remains a major challenge to disease control in the swine industry. Since NADC30-like PRRSV was first detected in China in 2013, it has become a particular concern because of its widespread circulation, frequent recombination, and variable pathogenicity. Here, three PRRSV isolates, SCABTC-202503, SCABTC-202505, and SCABTC-202506, were isolated and characterized. Whole-genome sequencing and phylogenetic analyses placed all three isolates within the NADC30-like L1C sublineage and identified characteristic discontinuous amino acid deletions in Nsp2. Recombination analyses indicated a shared NADC30-like genomic backbone, but distinct putative recombination patterns. Comparative GP5 analysis showed that SCABTC-202503 and SCABTC-202505 each harbored a single-amino-acid deletion at position 37 and multiple additional substitutions, whereas SCABTC-202506 encoded a full-length GP5 protein. In piglet challenge experiments, the three isolates showed different patterns of disease-associated changes, with variation in clinical scores, viral loads, cytokine levels, and the extent of lung lesions among the infected groups. However, because the isolates differed at multiple genomic sites, the association between the GP5 deletion and the observed phenotypic variation remains correlative. Establishing the specific contribution of the GP5 deletion will require infectious clone-based mutagenesis in a controlled genetic background. Collectively, these findings highlight the genetic and phenotypic heterogeneity of the NADC30-like PRRSV isolates examined and underscore the importance of continued surveillance of emerging variants.

**IMPORTANCE:**

Porcine reproductive and respiratory syndrome virus (PRRSV) remains a major pathogen affecting the swine industry because of its rapid evolution, frequent recombination, and variable pathogenicity. NADC30-like PRRSV has circulated widely in China and continues to diversify genetically. In this study, we obtained three recently circulating NADC30-like PRRSV isolates belonging to the L1C sublineage from clinical samples collected in China. We characterized their whole-genome sequences, phylogenetic relationships, putative recombination patterns, GP5 and Nsp2 sequence variations, and disease-associated changes in piglets. Under the conditions tested, the isolates exhibited genetic heterogeneity and varying degrees of clinical, virological, pathological, and inflammatory changes. These findings expand current knowledge of NADC30-like PRRSV diversity and underscore the importance of continued genomic surveillance for tracking emerging variants and informing future prevention and control strategies.

## INTRODUCTION

Porcine reproductive and respiratory syndrome (PRRS) is one of the most economically important viral diseases affecting the global swine industry (1, 2). It is characterized by reproductive failure in sows and respiratory disease in pigs of all ages and is frequently associated with growth retardation, increased mortality, and secondary infections. Given its broad impact across multiple production stages, PRRS remains a major challenge to the sustainable development of intensive swine production systems (3).

The causative agent of PRRS is porcine reproductive and respiratory syndrome virus (PRRSV), an enveloped, positive-sense RNA virus belonging to the genus Betaarterivirus within the family Arteriviridae (4). The viral genome is approximately 15 kb in length and comprises a 5′ untranslated region, multiple overlapping open reading frames (ORFs), and a 3′ poly(A) tail (1, 5). PRRSV is classified into two species, Betaarterivirus suid 1 (PRRSV-1; prototype strain LV) and Betaarterivirus suid 2 (PRRSV-2; prototype strain VR-2332), which share only approximately 60% nucleotide identity across the whole genome despite causing similar clinical manifestations (6–8). In China, PRRSV-2 has remained the predominant circulating species and has undergone extensive genetic diversification during prolonged field circulation.

During replication, the ORF1a and ORF1b regions at the 5′ end of the PRRSV genome are translated into the polyproteins pp1a and pp1ab, which are subsequently cleaved by viral proteases to produce multiple nonstructural proteins (9, 10). These nonstructural proteins assemble into the replication-transcription complex, which mediates viral genome replication and the synthesis of nested subgenomic mRNAs (9, 10). ORFs 2–7 at the 3′ end of the genome encode the structural proteins, which are translated from subgenomic mRNAs (11, 12). These structural proteins assemble with the nucleocapsid to form mature virions enclosed by a lipid envelope containing the membrane protein (M), the envelope protein (E), and the major envelope glycoprotein GP5 (13, 14). Because the viral RNA-dependent RNA polymerase lacks proofreading activity, and because discontinuous transcription and recombination further promote sequence variation, PRRSV exhibits substantial genetic plasticity, particularly in genomic regions encoding proteins involved in host interaction and antigenicity (15, 16).

Among the structural proteins of PRRSV, GP5, encoded by ORF5, is the major envelope glycoprotein and plays a key role in virion assembly and viral entry through interaction with the membrane protein M (9, 13, 17). The ectodomain of GP5 contains important antigenic determinants and is subject to strong host immune pressure, making it a hotspot for sequence variation (18–20). Previous studies have shown that GP5 diversity is primarily manifested as amino acid substitutions, which may alter antigenic properties and affect immune recognition (21, 22). In contrast, natural deletions in GP5 have rarely been reported in field isolates, unlike the frequent insertions and deletions observed in Nsp2 (23–25). Consequently, the molecular characteristics and biological significance of naturally occurring GP5 deletion variants remain poorly understood.

In recent years, NADC30-like PRRSV has become widely distributed in China and has emerged as one of the major circulating PRRSV lineages (26). Since 2013, NADC30-like strains have frequently recombined with classical PRRSV strains, highly pathogenic PRRSV, and other field strains, thereby generating substantial diversity in genomic architecture and biological characteristics (27). This lineage is typically characterized by a discontinuous 131-amino-acid deletion in Nsp2, which serves as a widely used molecular marker (28). In addition, substantial sequence variation has been observed in GP5 among NADC30-like strains (29). Taken together, these findings indicate ongoing diversification of NADC30-like PRRSV under field conditions.

In the present study, we isolated three NADC30-like PRRSV strains belonging to the L1C sublineage from clinical samples collected in Jiangxi and Sichuan provinces, China. Notably, two of these strains harbored a naturally occurring single-amino-acid deletion in GP5, along with multiple additional amino acid substitutions, whereas the third retained a GP5 sequence of typical length. We therefore combined whole-genome sequencing (WGS), phylogenetic, recombination, and amino acid variation analyses with piglet challenge experiments to compare their genomic characteristics and pathogenic properties. Collectively, these findings offer insight into the genomic complexity and pathogenic variation of NADC30-like PRRSV strains.

## MATERIALS AND METHODS

### Sample collection and processing

A total of 33 samples, including lung tissue, lymphoid tissue, and serum, were collected from three pig farms in Wanzai, Jiangxi Province, and Suining, Sichuan Province, China (Tables S1 and S2). Serum samples were filtered through 0.22-µm membranes and stored for subsequent analysis. Lung and lymphoid tissue samples were minced and ground into a fine powder in liquid nitrogen. An appropriate volume of Dulbecco’s modified Eagle medium (DMEM; Thermo Scientific, MA, USA) was then added, and the tissue powders were thoroughly homogenized. The homogenates were centrifuged at 4°C and 4,500 × *g* for 5 min, and the supernatants were collected for subsequent analysis. Total nucleic acids were extracted from tissue supernatants and serum samples using the TIANamp Virus DNA/RNA Kit (Tiangen, Beijing, China) according to the manufacturer’s instructions. cDNA was then synthesized using PrimeScript RT Master Mix (Takara, Beijing, China), and PRRSV was detected by real-time quantitative PCR (RT-qPCR) targeting the ORF7 gene (30).

### Virus isolation and identification

PRRSV-positive samples were used for virus isolation. MARC-145 cells at 80%–90% confluence in T25 flasks were washed twice with phosphate-buffered saline. Cells were inoculated with 1 mL sterile-filtered PRRSV-positive sample for 2 h at 37°C in 5% CO_2_. After adsorption, cells were maintained in DMEM containing 2% fetal bovine serum (Gibco, USA) for 4 days, and cytopathic effects (CPE) were monitored daily. When obvious CPE appeared, cultures were subjected to three freeze–thaw cycles at −80°C and centrifuged at 5,000 × *g* for 5 min. Supernatants were collected and stored at −80°C. The isolated virus was passaged 10 times, and viral RNA in each passage supernatant was quantified by RT-qPCR. Viral suspension from passage 10 was subjected to transmission electron microscopy (TEM) at Chengdu Lilai Biotechnology Co., Ltd. Viral titers were determined by TCID_50_ assay, and growth curves were generated. PRRSV infection was confirmed by indirect immunofluorescence assay (IFA) using a monoclonal antibody against PRRSV N protein (GeneTex, California, USA) and a FITC-conjugated secondary antibody (Proteintech, Wuhan, China). Purified viral supernatants were stored at −80°C.

### Genome sequencing and phylogenetic analysis

Viral suspensions from passage-10 virus stocks were subjected to WGS by Shanghai Tanpu Biotechnology Co., Ltd. For WGS analysis, clean reads were assembled *de novo* using metaSPAdes v.3.13.0 (31). Final scaffolds with a minimum contig length of 100 bp were searched against the NCBI Nucleotide database using MEGABLAST to identify PRRSV-related sequences. The WGS results were validated by RT-PCR using Phanta UniFi High-Fidelity DNA Polymerase (Vazyme, Nanjing, China) and primers designed with Primer Premier 5 based on the assembled sequences (Table S3). The resulting amplicons were subsequently subjected to Sanger sequencing at Sangon Biotech Co., Ltd. (Shanghai, China).

For phylogenetic analysis, 72 representative PRRSV reference strains were retrieved from GenBank (Table S4). Complete genome and ORF5 nucleotide sequences were aligned using MegAlign (DNASTAR Inc., USA) to calculate nucleotide sequence identities. The best-fit nucleotide substitution model for each data set was selected in MEGA 7.0 based on the lowest Bayesian Information Criterion value. Phylogenetic trees were constructed using the Maximum Likelihood (ML) method implemented in MEGA 7.0 (32). The General Time Reversible model with gamma-distributed rate variation and a proportion of invariant sites (GTR + G+ I) and the Kimura 2-parameter model with gamma-distributed rate variation and a proportion of invariant sites (K2 +G + I) were selected as the best-fit models for the complete genome and ORF5 data sets, respectively. Node support was assessed using 1,000 bootstrap replicates. The resulting phylogenetic trees were visualized and annotated using iTOL v.6.9 (https://itol.embl.de/) (33). For comparative amino acid analysis, ORF5 and Nsp2 amino acid sequences were aligned separately using MegAlign.

### Mutation and recombination analysis

To identify potential mutations and recombination events in the three isolates, representative PRRSV sequences from different lineages were retrieved from GenBank and aligned in MEGA. Because GP5 and Nsp2 are key determinants of PRRSV immunogenicity, replication, and pathogenesis, amino acid sequences of these proteins from the isolates and reference strains were aligned with the Clustal W algorithm in DNASTAR (DNASTAR Inc., Wisconsin, USA) to characterize sequence variation. Potential recombination events were initially screened in RDP4 and further evaluated using seven algorithms: RDP, GENECONV, BootScan, MaxChi, Chimaera, SiScan, and 3Seq (34–40). Recombination events were considered significant only if detected by at least three algorithms with *P* < 0.05. Putative recombination events were further verified in SimPlot (ReduSoft Ltd., Germany).

### Animal experiments

To assess the pathogenicity of the isolates, 12 clinically healthy piglets were obtained from Meishan Wanjiahao Co., Ltd. for challenge studies. All piglets were confirmed to be negative for PRRSV antigen, PRRSV antibodies, and other major porcine pathogens. Piglets were randomly assigned to four groups (*n* = 3 per group) and housed in separate isolation rooms. The three challenge groups were intranasally inoculated with 2 mL of viral suspension containing 2 × 10^5^ TCID_50_/mL of SCABTC-202506, SCABTC-202503, or SCABTC-202505, respectively, whereas the control group received an equal volume of DMEM. During the experiment, rectal temperature, clinical scores (41), and survival were recorded daily, and body weight was monitored to calculate average daily gain. Serum samples and nasal, throat, and anal swabs were collected at 1, 3, 5, 7, 9, 11, 13, and 14 days post-inoculation (dpi). At 15 dpi, all surviving piglets were euthanized with an intravenous overdose of sodium pentobarbital (Sinopharm, Beijing, China), and tissue samples, including the heart, liver, spleen, lung, kidney, tonsil, and lymph nodes, were collected. After necropsy, carcasses were temporarily stored in a designated freezer and subsequently disposed of by a qualified commercial service provider.

After fixation in 4% paraformaldehyde, lung tissues were processed for hematoxylin and eosin (H&E) staining and immunohistochemistry (IHC). For IHC, a polyclonal antibody against the PRRSV GP5 protein (Bioss, China) was used as the primary antibody. Viral loads in serum, swab, and tissue samples were quantified by RT-qPCR. Serum antibodies against the PRRSV N protein were qualitatively detected using the IDEXX PRRS 2XR enzyme-linked immunosorbent assay (ELISA) kit (IDEXX, USA). Serum inflammatory cytokine levels were measured using a commercial ELISA kit (Jianglai Biotechnology Co., Ltd., China).

### Statistical analysis

Statistical analyses were performed using two-way analysis of variance in GraphPad Prism 8.0.2 (San Diego, USA). A *P*-value <0.05 was considered statistically significant. Data are presented as mean ± standard deviation (SD), and all experiments were performed with at least three independent replicates. Figures were prepared using Adobe Illustrator 2020 (Adobe Inc., San Jose, CA, USA).

## RESULTS

### Virus isolation and identification

PRRSV-positive serum samples and tissue homogenate supernatants were inoculated into MARC-145 cells for virus isolation. After serial passage, three PRRSV isolates were successfully obtained and designated SCABTC-202503, SCABTC-202505, and SCABTC-202506.

**Fig 1:**
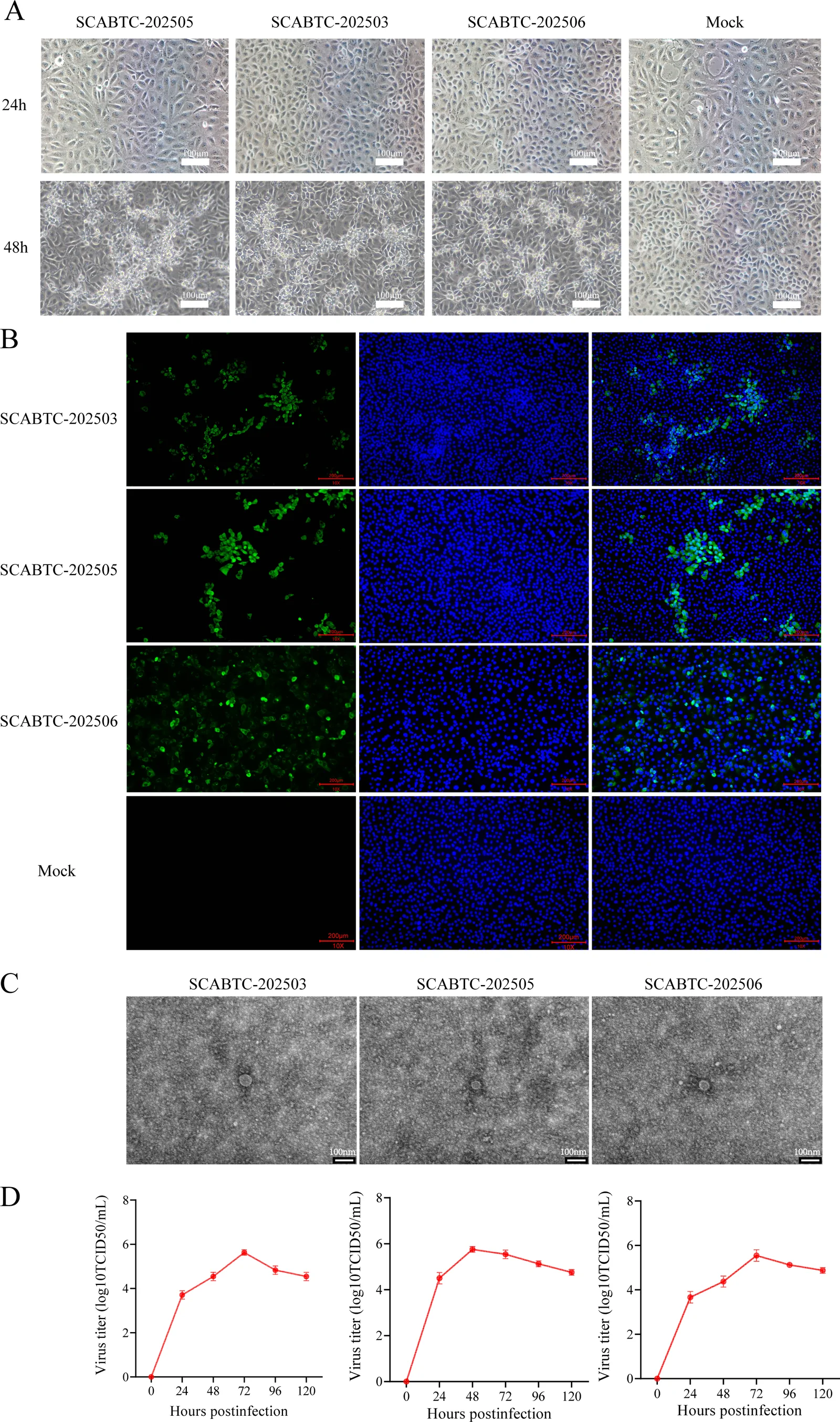
Infection and replication characteristics of the isolated PRRSV strains in MARC-145 cells. (**A**) CPE induced by the isolates at 24 and 48 hpi. (**B**) PRRSV N protein expression detected by IFA. (**C**) TEM images of virions. (**D**) Multi-step growth curves of the isolates in MARC-145 cells, with viral titers determined by the TCID_50_ assay. Scale bars: 100 μm in A, 200 μm in B, and 100 nm in C.

The three isolates were inoculated into MARC-145 cells to assess their cytopathic effects *in vitro*. At 24 h post-infection (hpi), no apparent CPE was observed in any of the infected groups (Fig. 1A). By 48 hpi, all three isolates induced typical PRRSV-associated CPE. Partial cell detachment was most pronounced in the SCABTC-202505-infected group, whereas no obvious cytopathic changes were observed in the mock-infected control group (Fig. 1A). IFA revealed specific green fluorescence signals in all infected groups, whereas no fluorescence was detected in the mock-infected control group (Fig. 1B). TEM further demonstrated that all three isolates exhibited typical PRRSV virion morphology (Fig. 1C).

The multistep growth curves of the three viruses in MARC-145 cells showed that viral titers increased progressively during the early phase of infection. SCABTC-202503 and SCABTC-202506 reached peak titers of 10^5.63^ and 10^5.54^ TCID_50_/mL at 72 hpi, respectively, whereas SCABTC-202505 reached a peak titer of 10^5.75^ TCID_50_/mL at 48 hpi, followed by a decline (Fig. 1D). This decline may be associated with the earlier and more evident CPE observed in SCABTC-202505-infected cells, although the contribution of virus-induced cell damage was not directly assessed in this study. These results further supported the successful isolation and characterization of SCABTC-202503, SCABTC-202505, and SCABTC-202506.

### Genome sequence and phylogenetic analysis

**Fig 2:**
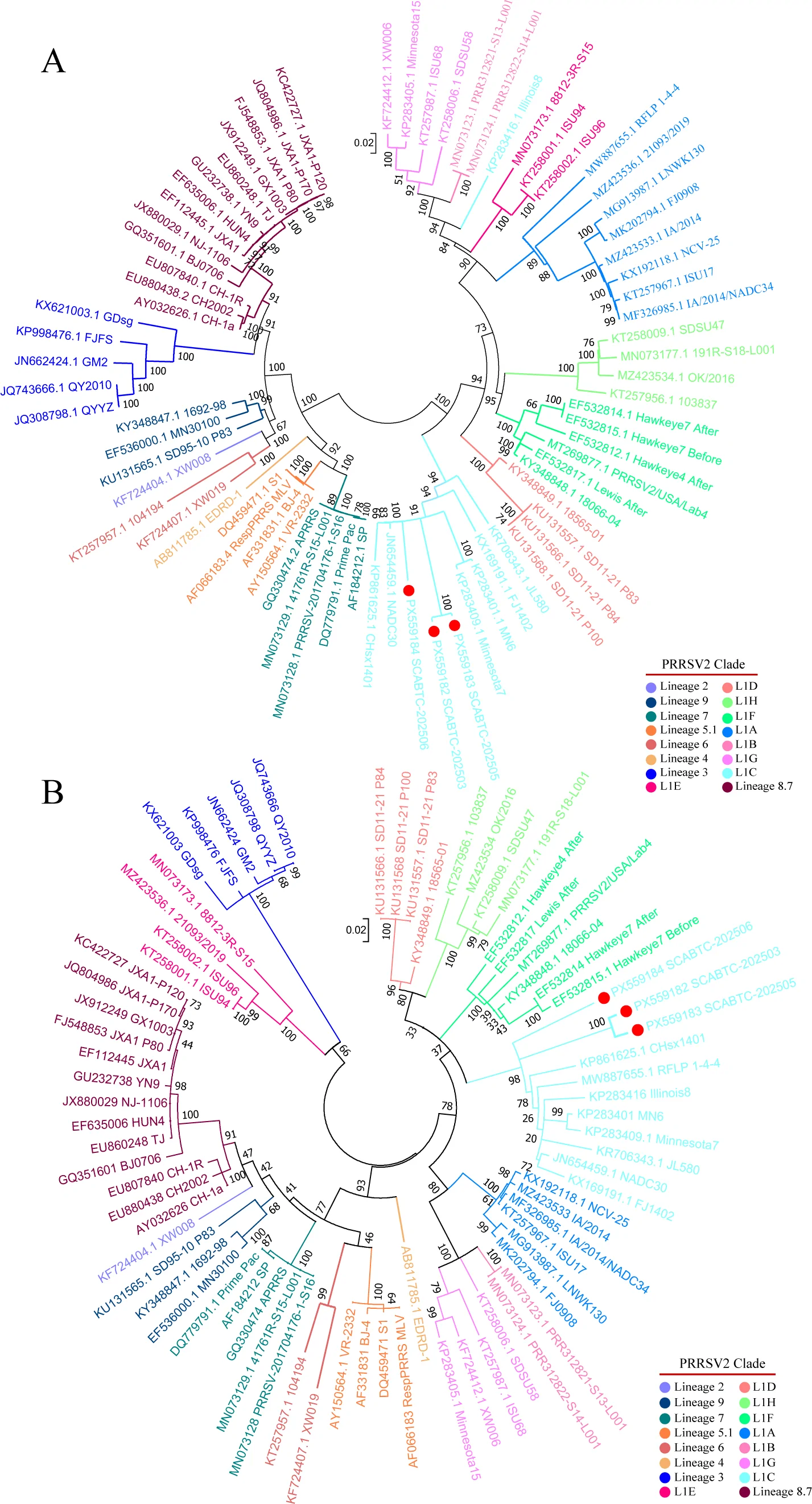
Phylogenetic analysis of the isolated PRRSV strains. (**A**) Phylogenetic tree based on complete-genome sequences. (**B**) Phylogenetic tree based on ORF5 sequences. Phylogenetic trees were reconstructed using the ML method implemented in MEGA 7.0, with GTR + G + I and K2 + G + I specified as the nucleotide substitution models for the complete-genome and ORF5 data sets, respectively. Node support was evaluated using 1,000 bootstrap replicates, and support values are shown at key nodes. Trees were visualized using iTOL v.6.9; different colors indicate distinct lineages, and the newly isolated strains are marked with red circles.

Complete genome sequences of the three newly isolated PRRSV strains were obtained by WGS and validated by Sanger sequencing. Standard sequencing metrics, including the proportion of aligned reads, mean depth, median depth, coverage breadth, and the proportions of contig regions with coverage depths >10× and >100×, are summarized in Table S5. The genome lengths of SCABTC-202503 and SCABTC-202505 were both 15,015 bp, whereas that of SCABTC-202506 was 15,017 bp. Following annotation with reference to previously reported PRRSV strains, the sequences were deposited in GenBank under accession numbers PX559182, PX559183, and PX559184, respectively. The corresponding raw WGS reads were deposited in the NCBI Sequence Read Archive (SRA) under BioProject PRJNA1512728, with SRA accession numbers SRR40164796, SRR40164795, and SRR40164794, respectively. Phylogenetic analyses based on the complete genome and ORF5 sequences consistently showed that all three isolates clustered within the L1C sublineage (Fig. 2A and B).

Among the compared reference strains, the highest whole-genome nucleotide identities of SCABTC-202503, SCABTC-202505, and SCABTC-202506 were observed with the NADC30 strain, with values of 91.1%, 91.2%, and 94.1%, respectively (Table S4). Sequence identities at the individual gene and deduced amino acid levels also varied between the three isolates and the NADC30 strain (Tables S6 and S7).

### Amino acid mutation analysis

**Fig 3:**
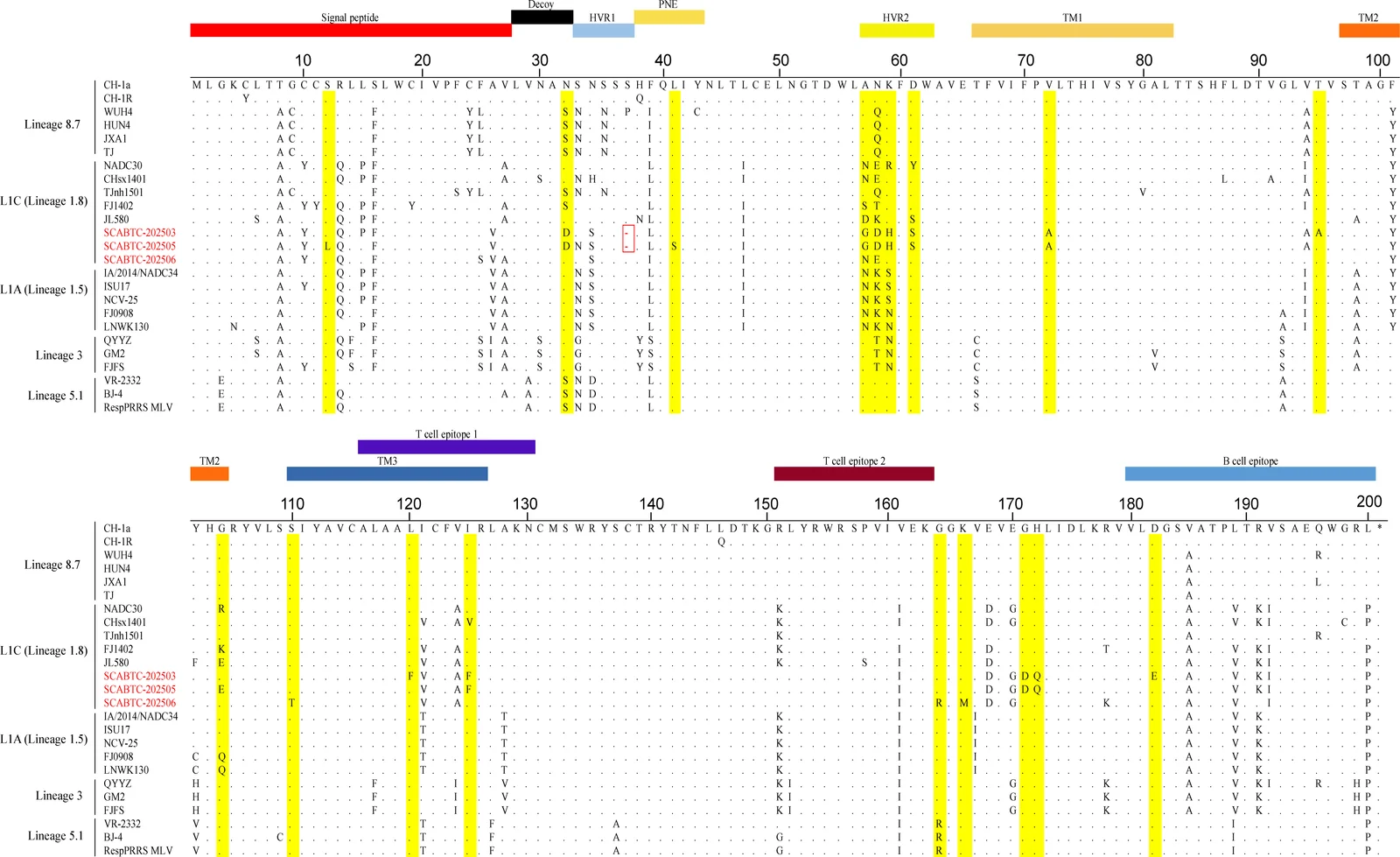
Alignment of ORF5 amino acid sequences. The red box indicates the amino acid deletion in SCABTC-202503 and SCABTC-202505, whereas yellow highlights indicate amino acid substitutions unique to the newly isolated strains.

**Fig 4:**
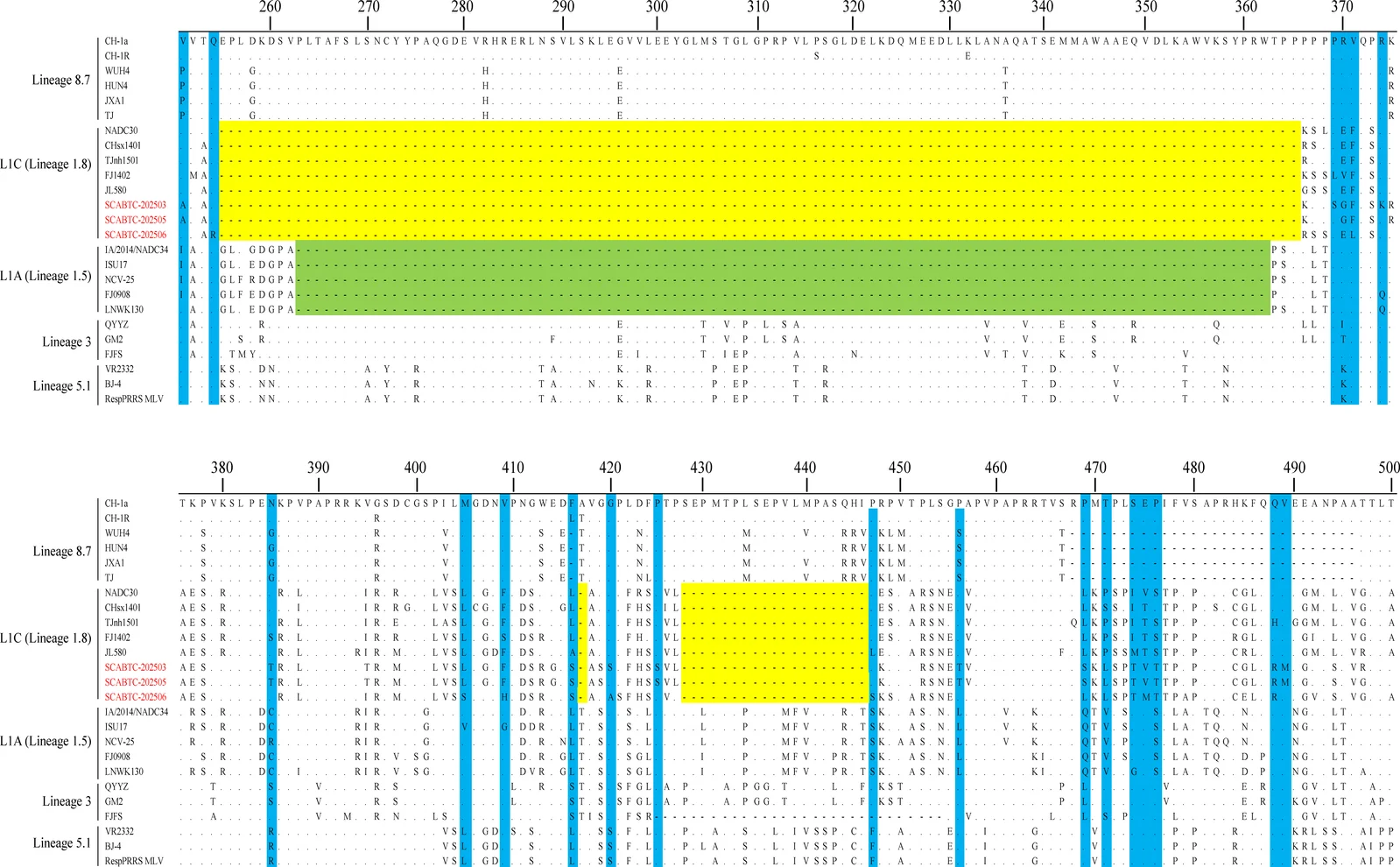
Alignment of Nsp2 amino acid sequences. Yellow highlights indicate the deletion specific to L1C, green highlights indicate the deletion specific to L1A, and blue highlights indicate amino acid substitutions identified in the isolates.

Among PRRSV proteins, GP5 and Nsp2 exhibit high genetic diversity and relatively low amino acid sequence conservation (42). Their high variability makes GP5 and Nsp2 important molecular markers for tracing PRRSV evolution and epidemiological patterns (43). Comparative analysis of ORF5 amino acid sequences showed that the three isolates harbored multiple amino acid substitutions in the signal peptide, decoy region, hypervariable region 2 (HVR2), transmembrane domains (TM1, TM2, and TM3), and T- and B-cell epitope regions. SCABTC-202503 and SCABTC-202505 shared several identical amino acid substitutions that were distinct from those of the reference strains, including N^32^→D^32^, A^57^→G^57^, N^58^→D^58^, K^59^→H^59^, D^61^→S^61^, V^72^→A^72^, I^125^→F^125^, G^171^→D^171^, and H^172^→Q^172^. In addition, strain-specific amino acid substitutions were identified in SCABTC-202503 (L^120^→F^120^, D^182^→E^182^), SCABTC-202505 (S^12^→L^12^, L^41^→S^41^, G^104^→E^104^), and SCABTC-202506 (N58→E58, S110→T110, K166→M166, G164→R164) (Fig. 3). Notably, comparative GP5 sequence analysis revealed that SCABTC-202503 and SCABTC-202505 each harbored a single-amino-acid deletion at GP5 position 37, along with multiple amino acid substitutions elsewhere in the protein (Fig. 3). Comparative Nsp2 amino acid sequence analysis also showed that all three strains exhibited the discontinuous Nsp2 deletion pattern characteristic of NADC30-like PRRSV and carried multiple additional amino acid substitutions (Fig. 4).

We also performed Sanger sequencing of the complete GP5 and Nsp2 sequences in the first-passage cell-culture virus stocks and compared these sequences with those from the passage-10 virus stocks used in this study. No mutations were detected in the complete GP5 and Nsp2 sequences of the passage-10 virus stocks when compared with those of the first-passage cell-culture virus stocks, indicating that the passage-10 viruses retained the GP5 and Nsp2 sequence characteristics of the early-passage isolates. The sequencing results are presented in Table S8.

### Recombination analysis

**Fig 5:**
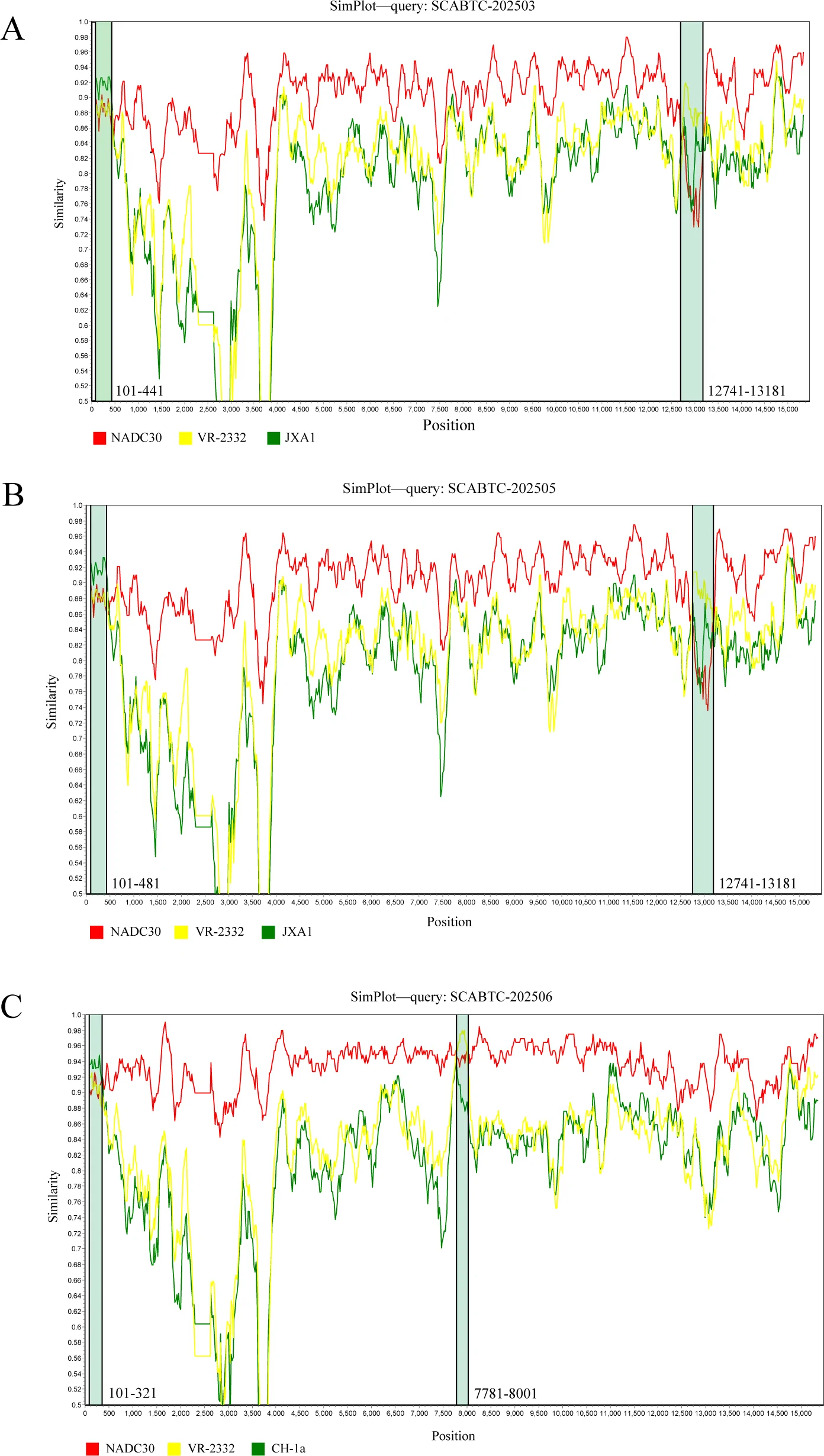
Recombination analysis of the isolated PRRSV strains. SimPlot was used to analyze putative recombination patterns in (**A**) SCABTC-202503, (**B**) SCABTC-202505, and (**C**) SCABTC-202506. Green regions indicate putative recombinant regions, and adjacent numbers indicate their corresponding breakpoint coordinates.

Putative recombination events in the three isolates were analyzed using representative reference strains, including QYYZ, JXA1, IA/2014/NADC34, NADC30, VR-2332, and CH-1a. Initial screening with RDP4 identified putative recombination signals in all three isolates (Table S9), and SimPlot was subsequently used to evaluate these signals and visualize the inferred recombination patterns (Fig. 5A through C). SCABTC-202503 and SCABTC-202505 showed overall genomic similarity to NADC30, whereas two genomic regions were more similar to VR-2332 and JXA1, suggesting more complex putative recombination patterns. In contrast, SCABTC-202506 contained fewer putative recombinant regions and showed greater overall similarity to NADC30; NADC30, VR-2332, and CH-1a were inferred as candidate parental strains in the recombination analysis. Together, these results suggest that the three isolates differ not only in GP5 sequence features but also in their overall genomic architecture and putative recombination profiles.

### Clinical signs

**Fig 6:**
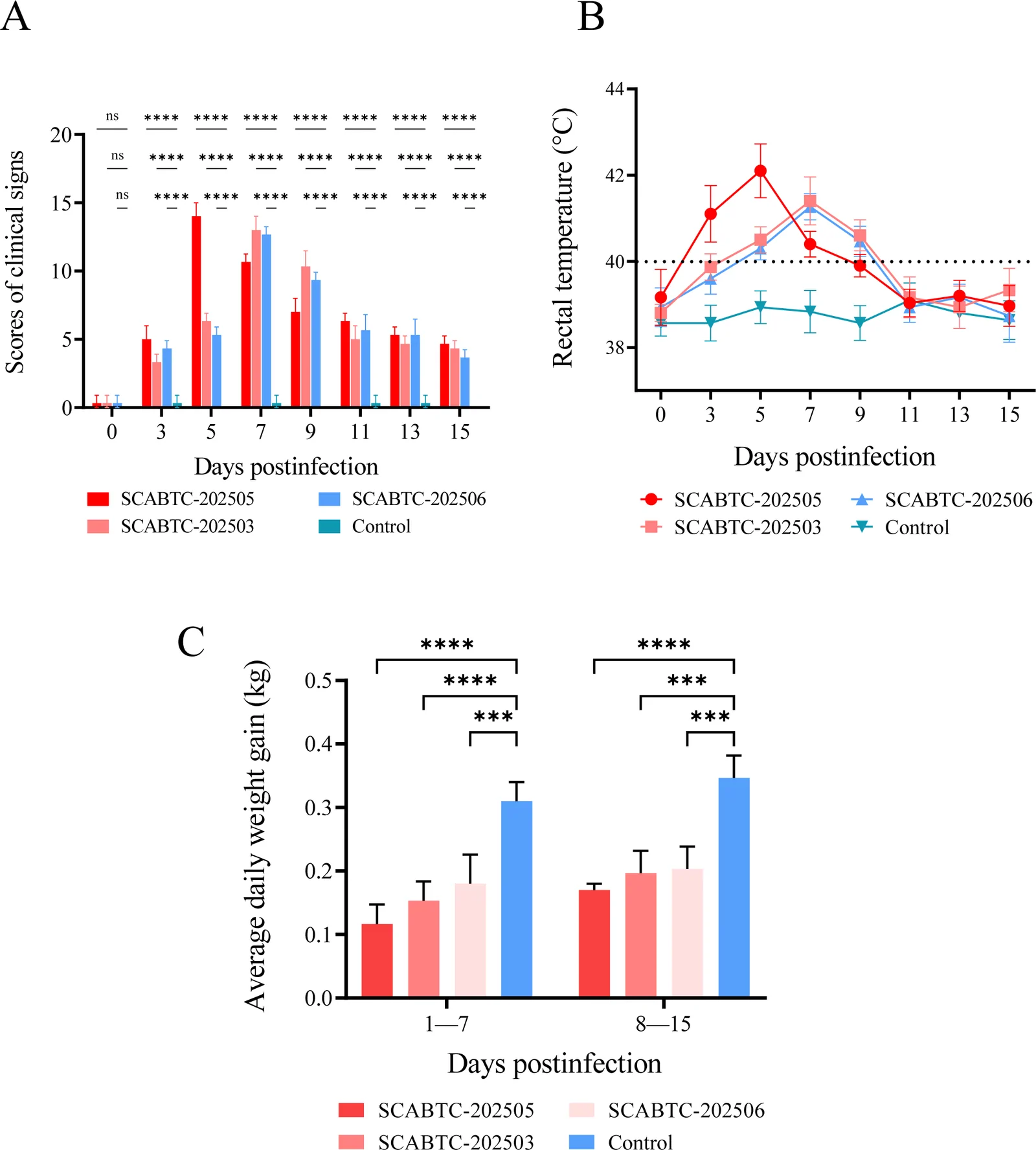
Clinical manifestations in piglets following infection. (**A**) Clinical scores. (**B**) Rectal temperature. (**C**) Average daily gain. Data are presented as mean ± SD. ns, not significant; *P < 0.05; **P < 0.01; ***P < 0.001; ****P < 0.0001. SD, standard deviation.

To evaluate clinical manifestations and growth performance following infection with the three PRRSV isolates, clinical signs, rectal temperature, and average daily gain were assessed. All infected piglets developed clinical signs, including fever and dyspnea. Clinical scores were elevated in all infected groups compared with the control group, with numerically higher scores in the SCABTC-202505-infected group at several time points (Fig. 6A). Rectal temperature monitoring showed that piglets infected with SCABTC-202505 had temperatures above 40°C from 3 to 7 dpi, with the highest values recorded at 5 dpi. Piglets infected with SCABTC-202503 or SCABTC-202506 showed increased rectal temperatures from 3 dpi, with values remaining above 40°C from 5 to 9 dpi and reaching their highest levels at 7 dpi in both groups (Fig. 6B). In addition, average daily gain during both the 1–7 and 8–15 dpi periods was lower in all three infected groups than in the control group (Fig. 6C), suggesting lower growth performance in infected piglets under the conditions tested. No apparent clinical signs were observed in the control group throughout the experiment, and all control piglets survived.

### Viremia, viral shedding, tissue distribution, and antibody response

**Fig 7:**
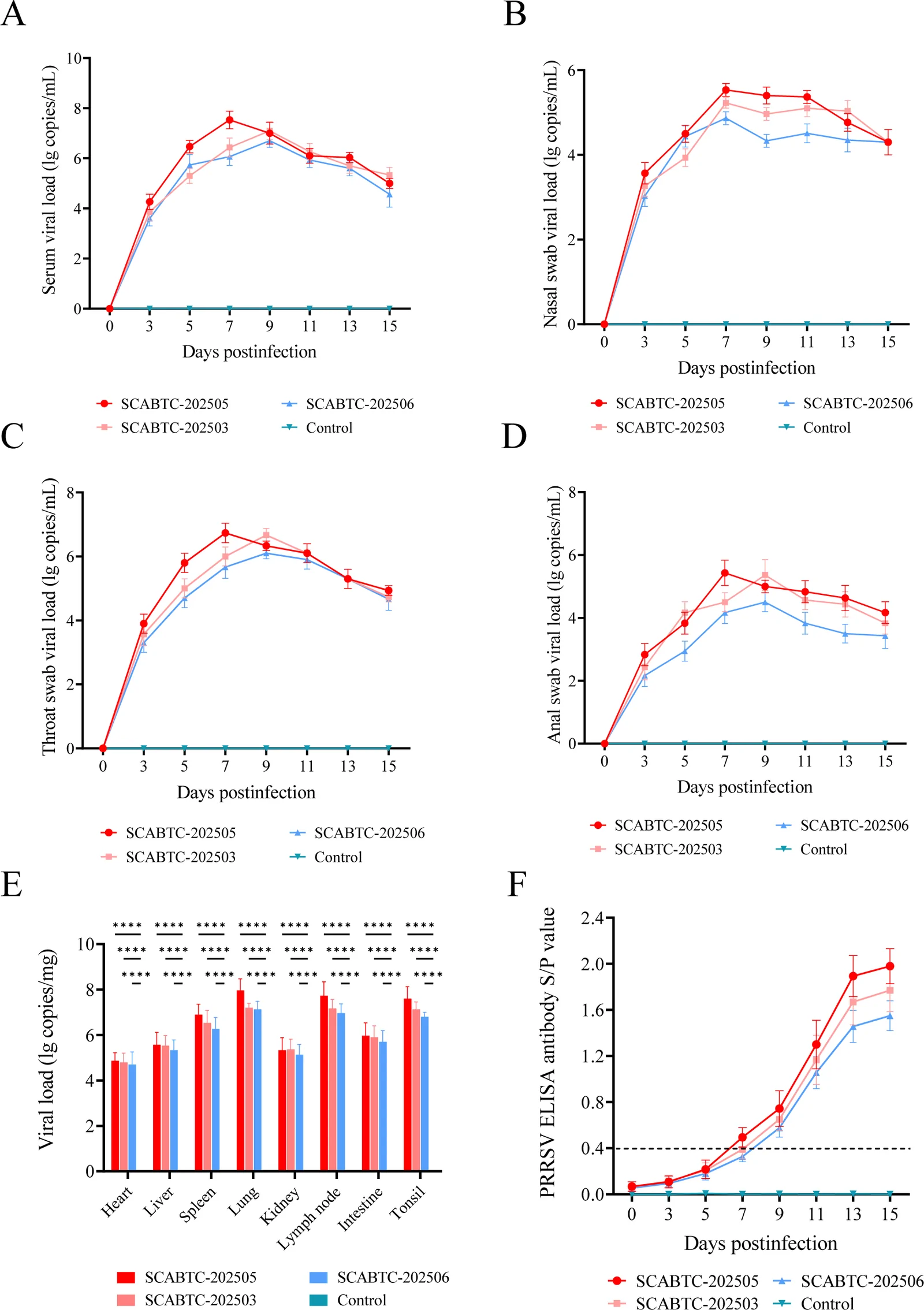
Changes in viremia, viral shedding, tissue distribution, and antibody responses in piglets following infection. (**A**) Viral load in serum. (**B**) Viral shedding in nasal swabs. (**C**) Viral shedding in throat swabs. (**D**) Viral shedding in anal swabs. (**E**) Viral loads in different tissues. (**F**) PRRSV N-specific antibody responses in each group. Data are presented as mean ± SD. ns, not significant; *P < 0.05; **P < 0.01; ***P < 0.001; ****P < 0.0001. SD, standard deviation.

To evaluate viremia and viral shedding patterns, serum samples and nasal, throat, and anal swabs were collected from piglets at 0, 3, 5, 7, 9, 11, 13, and 15 dpi for viral load quantification. In the SCABTC-202505-infected group, viral loads in serum and swab samples increased until 7 dpi, when they reached their highest observed levels, and then gradually declined. Similar overall temporal patterns were observed in piglets infected with SCABTC-202503 or SCABTC-202506, with viral loads differing numerically among the infected groups at several time points (Fig. 7A through D). In addition, viral RNA was detected in multiple tissues from infected piglets, with relatively high viral loads observed in the lungs and lymph nodes. No viral RNA was detected in samples from the control group (Fig. 7E).

Detection of PRRSV N protein-specific antibodies showed that all piglets in the infected groups seroconverted by 7 dpi (S/P ≥ 0.4). Among the infected groups, piglets infected with SCABTC-202505 showed relatively higher antibody levels at 7, 9, and 11 dpi than piglets infected with SCABTC-202503 or SCABTC-202506. Thereafter, serum antibody levels continued to increase in all infected groups over the course of infection. In contrast, piglets in the control group remained seronegative throughout the experiment (Fig. 7F).

### Pathological lesions

**Fig 8:**
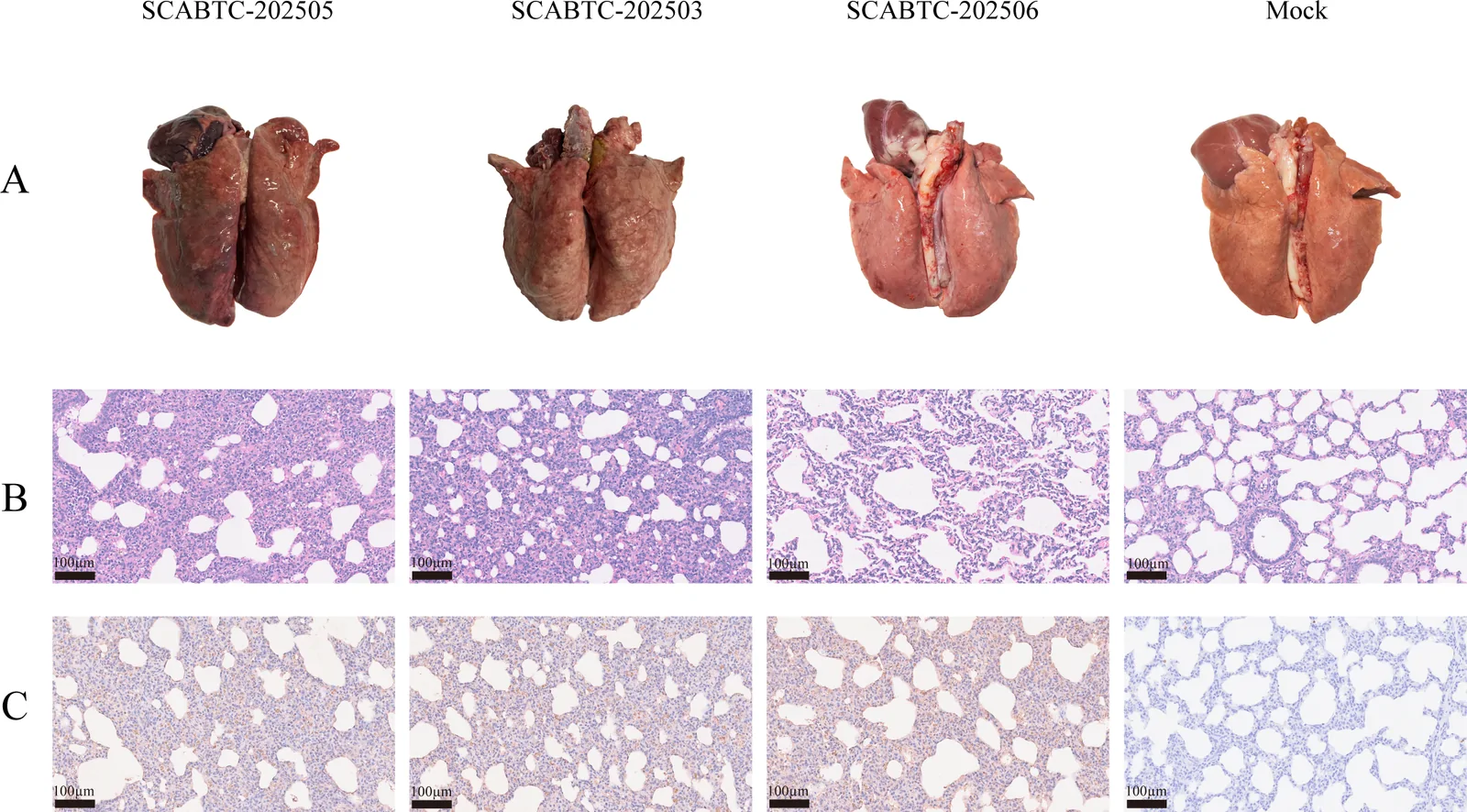
Gross lesions, histopathological changes, and immunohistochemical analysis of piglet lungs following infection. (**A**) Gross lung lesions. (**B**) Histopathological changes assessed by H&E staining. (**C**) Immunohistochemical detection of PRRSV GP5 protein. H&E-stained and IHC images were acquired at 200 × magnification. Scale bar, 100 μm. In the IHC images, brown-yellow granules indicate positive staining for the PRRSV GP5 protein. H&E, hematoxylin and eosin; IHC, immunohistochemistry.

Gross pathological examination revealed lung lesions of varying extent in piglets from all three infected groups. Gross lung lesions appeared more extensive in piglets infected with SCABTC-202505 and were characterized by incomplete lung collapse, diffuse or multifocal dark red to purplish-red discoloration, and apparent congestion, hemorrhage, edema, and focal consolidation (44). Similar gross lesions were also observed in piglets infected with SCABTC-202503. In piglets infected with SCABTC-202506, gross lesions were generally similar in type to those described above but appeared less pronounced overall. In contrast, no apparent gross lesions were observed in the lungs of control piglets (Fig. 8A).

Histological analysis showed that lung tissue sections from all three infected groups exhibited inflammatory cell infiltration, alveolar epithelial hyperplasia, alveolar septal thickening, and capillary hemorrhage, whereas no apparent histopathological changes were observed in the control group (Fig. 8B). IHC staining further revealed brownish-yellow immunopositive signals in lung tissue sections from all three infected groups, whereas no immunopositive signal was detected in the control group (Fig. 8C). Overall, lung lesions appeared more pronounced in the SCABTC-202505-infected group and relatively milder in the SCABTC-202506-infected group under the conditions tested.

### Cytokine determination

**Fig 9:**
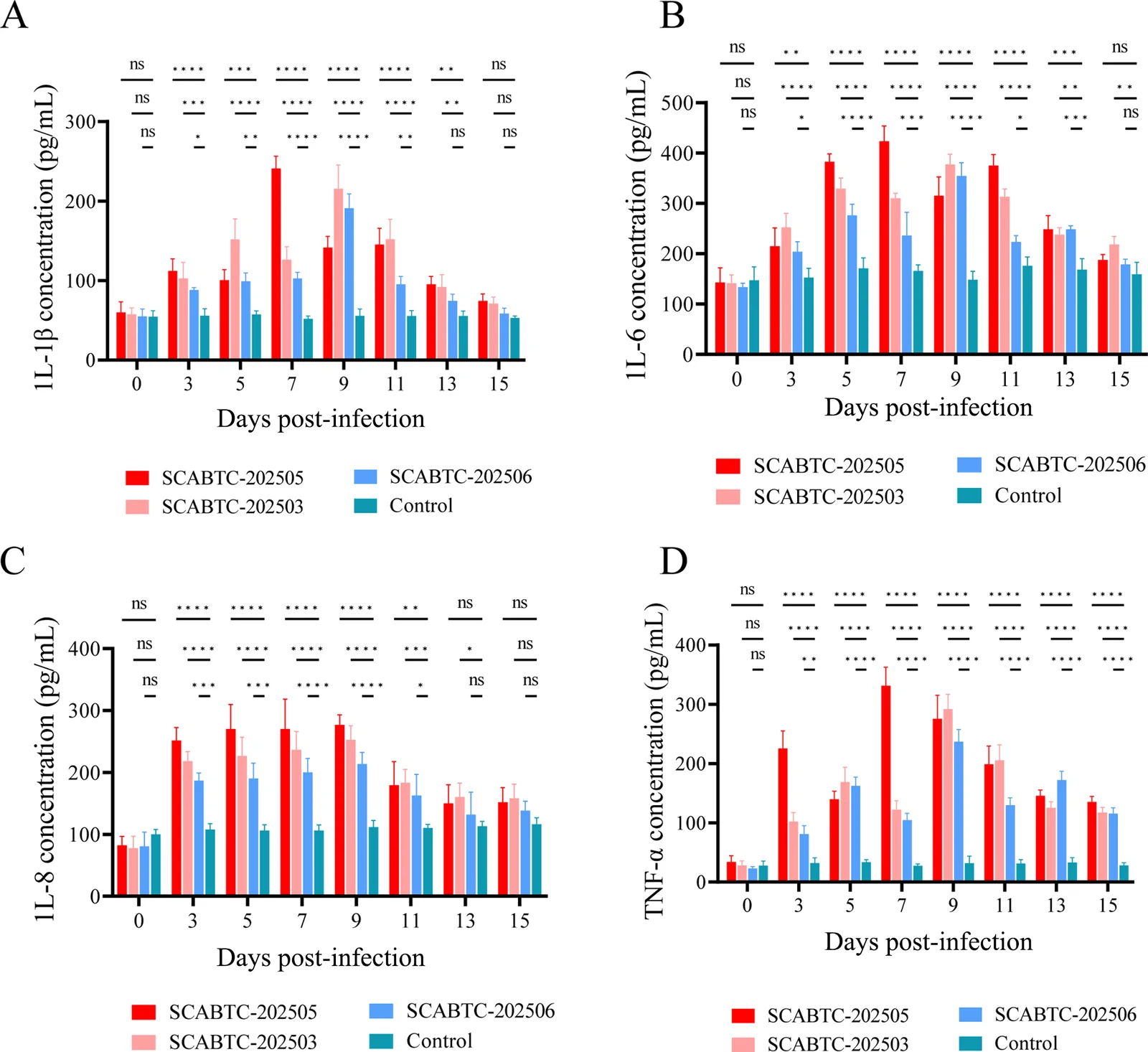
Changes in serum cytokine levels in piglets. Serum levels of (**A**) IL-1β, (**B**) IL-6, (**C**) IL-8, and (**D**) TNF-α were measured by ELISA. Data are presented as mean ± SD. ns, not significant; *P < 0.05; **P < 0.01; ***P < 0.001; ****P < 0.0001.

To evaluate inflammatory responses following infection with the three PRRSV isolates, temporal changes in serum IL-1β, IL-6, IL-8, and TNF-α levels were monitored from 0 to 15 dpi (Fig. 9A through D). Serum levels of these cytokines increased to varying degrees in all infected groups and were generally higher than those in control piglets. The magnitude and temporal patterns of cytokine responses varied among the infected groups, with numerically higher levels observed in piglets infected with SCABTC-202505 or SCABTC-202503 at several time points. Serum levels of most cytokines were elevated from 3 to 11 dpi, reached their highest observed values during the middle phase of infection, and then gradually declined. These results suggest that infection with the three isolates was associated with different cytokine-response patterns under the conditions tested.

## DISCUSSION

Previous studies have shown that the epidemiology and evolution of PRRSV in China have followed distinct stage-specific patterns (45, 46). Following the emergence of highly pathogenic PRRSV (HP-PRRSV; lineage 8) in 2006, this lineage remained dominant in Chinese swine populations for an extended period (47). Although the widespread use of modified live vaccines subsequently alleviated the epidemic to some extent, the high variability of the PRRSV RNA genome enabled continued immune evasion through ongoing mutation and frequent recombination, thereby progressively reducing control efficacy (48, 49). In recent years, multiple molecular epidemiological studies have shown that NADC30-like PRRSV has replaced previously dominant strains in many regions of China and become one of the major circulating lineages (26). This lineage exhibits substantial genetic plasticity and readily recombines with classical strains, HP-PRRSV, and vaccine-related strains, thereby generating a highly heterogeneous viral population in terms of genomic architecture and biological characteristics (27, 50).

In this study, three PRRSV strains were isolated and designated SCABTC-202503, SCABTC-202505, and SCABTC-202506. Phylogenetic analyses based on complete-genome and ORF5 sequences showed that all three isolates belonged to the NADC30-like L1C sublineage. Whole-genome sequence comparisons revealed that the highest nucleotide identities of SCABTC-202503, SCABTC-202505, and SCABTC-202506 to representative L1C strains were 91.1%, 91.2%, and 94.1%, respectively. Notably, the GP5 proteins of SCABTC-202503 and SCABTC-202505 each contained a naturally occurring single-amino-acid deletion, whereas SCABTC-202506 retained a GP5 protein of typical length. Further analysis showed that SCABTC-202503 and SCABTC-202505 harbored multiple amino acid substitutions across several functionally relevant regions, including the signal peptide, decoy epitope, PNE, HVR2, transmembrane domains, and predicted T- and B-cell epitopes, whereas SCABTC-202506 showed fewer amino acid variations. These findings suggest that substantial sequence divergence can occur even among isolates within the same sublineage. Although the biological significance of naturally occurring GP5 deletions remains incompletely understood, the coexistence of GP5 deletions and broader sequence variation in SCABTC-202503 and SCABTC-202505 suggests that field-circulating NADC30-like PRRSV strains may be undergoing continuous genetic diversification.

The coexistence of different PRRSV lineages in the field can create favorable conditions for viral recombination, thereby increasing genomic diversity and facilitating the emergence of novel epidemic strains (15, 51). Recombination analysis suggested that SCABTC-202503 and SCABTC-202505 both had an NADC30-like PRRSV genomic backbone but showed relatively high sequence similarity to VR-2332 and JXA1 in two genomic regions, suggesting more complex putative recombination patterns involving multiple candidate parental strains. In contrast, SCABTC-202506 contained fewer putative recombinant regions; NADC30, VR-2332, and CH-1a were inferred as candidate parental strains, suggesting a more limited putative recombination profile. Such recombination patterns may be associated with changes in viral replication, virulence, and immune evasion (10, 52).

Together, the animal challenge experiments and immunopathological analyses suggest that the three NADC30-like PRRSV isolates exhibited different patterns of disease-associated changes despite belonging to the same L1C sublineage. Under the conditions tested, piglets infected with SCABTC-202505 showed numerically higher clinical scores and cytokine levels at several time points, generally higher viral loads, and more evident pulmonary changes. Piglets infected with SCABTC-202503 also showed disease-associated changes across these measures, whereas those infected with SCABTC-202506 showed milder changes overall but still developed typical PRRSV-associated clinical signs. In all infected groups, serum IL-6, TNF-α, IL-1β, and IL-8 levels increased to varying degrees, and the cytokine response patterns were generally consistent with the clinical and pathological findings. Although SCABTC-202505 and SCABTC-202503 both carried a single-amino-acid deletion at GP5 position 37, the phenotypic variation observed among the infected groups occurred in the context of multiple other genomic differences and distinct putative recombination profiles. Accordingly, any association between the GP5 deletion and the observed phenotypic differences should be interpreted as correlative rather than causal, and the present data do not support attributing these differences specifically to the GP5 deletion. Functional validation using infectious clone-based mutagenesis in a controlled genetic background will be required to determine whether this deletion contributes to viral replication, immune modulation, or pathogenicity (17, 53, 54).

This study has several limitations. First, only three naturally occurring isolates were compared; therefore, the observed phenotypic differences may reflect differences at multiple genomic sites rather than the effect of any single mutation. Second, the animal experiments were conducted with a limited sample size (*n* = 3 per group), which may have reduced the statistical power for detecting between-group differences. Accordingly, the observed differences in clinical scores, rectal temperatures, and cytokine responses should be interpreted cautiously and warrant confirmation in larger animal cohorts. Third, infectious clone-based mutagenesis was not performed to determine the specific contribution of the GP5 deletion in a controlled genetic background; therefore, no causal relationship between the GP5 deletion and the observed phenotypic differences can be established from the present study. Finally, although Sanger sequencing confirmed that the complete GP5 and Nsp2 sequences of the passage-10 virus stocks were identical to those of the first-passage cell-culture virus stocks, potential passage-associated changes in other genomic regions cannot be excluded. Thus, associations between the genomic characteristics of the isolates and their disease-associated phenotypes should be interpreted in light of this limitation.

Overall, this study characterized three NADC30-like L1C PRRSV isolates with distinct genomic features, putative recombination profiles, and disease-associated phenotypes. SCABTC-202503 and SCABTC-202505 carried a single-amino-acid deletion at GP5 position 37 and showed greater genome-wide divergence from representative NADC30-like isolates than SCABTC-202506. Under the conditions tested, the three isolates exhibited different patterns of disease-associated changes, with variation in clinical, virological, inflammatory, and pathological measures among the infected groups. Because the isolates differed at multiple genomic sites, the contribution of any single genetic change cannot be resolved from the present comparisons and will require functional validation in a controlled genetic background. These findings expand current knowledge of the genetic and phenotypic diversity of NADC30-like PRRSV and underscore the importance of continued surveillance of emerging variants.

## Data Availability

The complete genome sequences of SCABTC-202503, SCABTC-202505, and SCABTC-202506 have been deposited in GenBank under accession numbers PX559182, PX559183, and PX559184, respectively. The corresponding raw WGS reads have been deposited in the NCBI Sequence Read Archive (SRA) under BioProject PRJNA1512728, with accession numbers SRR40164796, SRR40164795, and SRR40164794, respectively. All other data supporting the findings of this study are available from the corresponding author upon reasonable request.

## SUPPLEMENTAL MATERIAL

Supplemental tables spectrum.01220-26-s0001.pdf Tables S1 to S9.

ASM does not own the copyrights to Supplemental Material that may be linked to, or accessed through, an article. The authors have granted ASM a non-exclusive, world-wide license to publish the Supplemental Material files. Please contact the corresponding author directly for reuse.
