# Supplementary material for: Isolation, identification, and pathogenicity comparison of three NADC30-like PRRSV variants with distinct mutational patterns
Source: Microbiol Spectr. 2026 Oct 13;14(11):e01220-26. doi: 10.1128/spectrum.01220-26 (PMC13729182; doi:10.1128/spectrum.01220-26)
Supplement: Supplementary file 1 [file spectrum.01220-26-s0001.pdf]

Supplementary Table S1 Information on PRRSV Samples Collected from Sichuan Province

Sample no.	Specimen	CT	Judgment (+/−)	Collection area	Positive rate
NO.1	Lung	15.68	+	Sichuan Province	73.33%
NO.2	Lung	12.26	+		
NO.3	Serum	20.36	+		
NO.4	Serum	23.54	+		
NO.5	Serum	17.65	+		
NO.6	Serum	Undetermined	−		
NO.7	lymphoid	14.66	+		
NO.8	lymphoid	16.65	+		
NO.9	lymphoid	Undetermined	−		
NO.10	lymphoid	27.59	+		
NO.11	Lung	34.67	−		
NO.12	Lung	29.36	+		
NO.13	Lung	31.23	−		
NO.14	Lung	25.69	+		
NO.15	lymphoid	19.65	+		

CT<31 is considered a positive test result, while CT≥31 is considered a negative test result.

The symbol ‘+’ denotes a positive test result, while ‘-’ indicates a negative test result.

Supplementary Table S2 Information on PRRSV Samples Collected from Jiangxi Province

Sample no.	Specimen	CT	Judgment (+/−)	Province	Positive rate
NO.1	lymphoid	17.42	+	Jiangxi Province	72%
NO.2	lymphoid	25.67	+		
NO.3	Serum	Undetermined	−		
NO.4	Serum	21.34	+		
NO.5	Lung	27.56	+		
NO.6	Lung	19.12	+		
NO.7	Lung	15.48	+		
NO.8	Lung	23.77	+		
NO.9	lymphoid	11.93	+		
NO.10	lymphoid	26.05	+		
NO.11	Serum	14.68	+		
NO.12	Serum	22.41	+		
NO.13	Serum	32.87	−		
NO.14	Serum	Undetermined	−		
NO.15	Lung	33.45	−		
NO.16	Lung	32.98	−		
NO.17	Lung	20.36	+		
NO.18	Lung	19.64	+		

CT<31 is considered a positive test result, while CT≥31 is considered a negative test result.

The symbol '+' denotes a positive test result, while '-' indicates a negative test result.

Supplementary Table 3 Specific amplification primers designed based on the isolate sequences

Primer	Sequence information
F1	ATGACGTATAGGTGTTGGCTCT
R1	TTTCATCCAATTGGGCATACGC
F2	CGCAGGGCAGGTGCAAAAAGTG
R2	GCCAGTTCTCTCGCACAGCCTG
F3	TACGGAAATACCTGTTTGCCCA
R3	AAGGGCGCAGCCATTCCAGGTG
F4	TCTCCCAGATCTCGAAGCTTAC
R4	AATTTCGGCCGCATGGTTCTCG
GP5-F	GAATGTTTAGATATGTTGGGGAAATGC
GP5-R	CATTGCAAAAGTCATCTATGGACGAC
Nsp2-F	GCAATCGTCAACCATATAACACACTCT
Nsp2-R	GTGCCCACCGAGGTCATGTAAAT

Supplementary Table S4. Nucleotide sequence identity analysis of whole-genome and ORF5 genes between the isolates and reference PRRSV strains

Strain name	Lineage	GenBank accession number	SCABTC-202503		SCABTC-202505		SCABTC-202506	
			Complete genome identity (%)	ORF5 gene identity (%)	Complete genome identity (%)	ORF5 gene identity (%)	Complete genome identity (%)	ORF5 gene identity (%)
ISU17	L1A	KT257967.1	83.1	85.2	83.2	84.7	84.2	86.6
NCV-25	L1A	KX192118.1	83	85.4	83.1	84.6	84.1	85.7
IA/2014/NADC34	L1A	MF326985.1	83.4	85.2	83.4	84.7	84.5	86.6
LNWK130	L1A	MG913987.1	83.1	84.2	83.1	84.1	84	85.1
FJ0908	L1A	MK202794.1	83	84.4	83.1	84.6	84.1	85.9
RFLP_1-4-4	L1A	MW887655.1	83.6	90.5	83.7	90.5	84.8	90.7
IA/2014	L1A	MZ423533	83.1	84.6	83.1	84.1	84.2	85.9
21093/2019	L1A	MZ423536.1	82.8	84.2	82.3	84.2	83.2	85.6
PRR312821-S13-L001	L1B	MN073123.1	83.6	85.1	83.7	84.9	84.8	84.9
PRR312822-S14-L001	L1B	MN073124.1	83.6	85.1	83.7	84.9	84.8	84.9
NADC30	L1C	JN654459.1	91.1	91.2	91.2	91	94.1	91.5
MN6	L1C	KP283401	88.3	91	88.4	90.5	90.9	90.5
Minnesota7	L1C	KP283409.1	88.2	90.4	88.3	89.9	90.7	90
Illinois8	L1C	KP283416	85.4	88.6	85.5	88.4	87.3	90.2
CHsx1401	L1C	KP861625.1	89.5	89.2	89.6	89	92.2	90
JL580	L1C	KR706343.1	87.9	89.1	88	89.2	89.6	89.7
FJ1402	L1C	KX169191.1	88.9	89.6	88.9	89.7	91.1	90.7
SD11-21_P83	L1D	KU131557.1	83	87.1	83	86.9	84.1	87.4
SD11-21_P84	L1D	KU131566.1	83	87.1	83	86.9	84.1	87.4
SD11-21_P100	L1D	KU131568	83	87.1	83	86.9	84.1	87.4
18565-01	L1D	KY348849.1	84.2	88.1	84.2	87.6	85.8	88.2
ISU94	L1E	KT258001.1	82.7	83.7	82.8	83.7	83.9	85.4
ISU96	L1E	KT258002.1	82.9	83.7	82.9	83.7	84	85.4
8812-3R-S15	L1E	MN073173.1	81.4	82.9	81.6	83.4	82.7	84.6
Hawkeye4_After	L1F	EF532812.1	82.2	88.4	82.3	87.6	83.3	86.4
Hawkeye7_After	L1F	EF532814	82	86.2	82.1	85.7	83.2	85.9
Hawkeye7_Before	L1F	EF532815.1	82	85.6	82.1	85.4	83.1	85.6
Lewis_After	L1F	EF532817	82.5	87.4	82.5	86.6	83.6	87.1
18066-04	L1F	KY348848.1	84.6	87.1	84.7	86.6	85.7	87.1
PRRSV2/USA/Lab4	L1F	MT269877.1	83.7	86.6	83.8	85.7	84.9	85.1
XW006	L1G	KF724412.1	83.3	84.6	83.4	84.4	85	84.1
Minnesota15	L1G	KP283405.1	83.9	84.6	84	84.4	85.7	84.7
ISU68	L1G	KT257987.1	82.9	84.1	83	84.2	84.3	83.9
SDSU58	L1G	KT258006.1	82.7	84.2	82.8	83.9	83.9	84.2
103837	L1H	KT257956.1	83.6	86.2	83.6	85.7	84.6	85.1
SDSU47	L1H	KT258009.1	83.2	84.9	83.3	84.1	84.4	85.4

191R-S18-L001	L1H	MN073177.1	83.1	84.9	83.2	83.9	84.3	85.6
OK/2016	L1H	MZ423534	83.2	84.2	83.3	83.3	84.4	85.2
XW008	Lineage 2	KF724404.1	78.9	84.1	79	84.7	79.5	85.1
QYYZ	Lineage 3	JQ308798	77.9	82.4	78.1	82.4	78.8	83.1
QY2010	Lineage 3	JQ743666	77.9	82.4	78.1	82.4	78.8	83.1
FJFS	Lineage 3	KP998476	78.7	82.4	78.7	82.4	79.5	83.1
GDsg	Lineage 3	KX621003	78.6	81.8	78.8	82.4	79.5	83.1
GM2	Lineage 3	JN662424	78.1	82.1	78.2	82.1	79.1	82.8
EDRD-1	Lineage 4	AB811785.1	80.1	85.9	80.1	85.4	81	87.1
RespPRRS_MLV	Lineage 5.1	AF066183	81.3	84.2	81.4	84.4	82.6	85.2
BJ-4	Lineage 5.1	AF331831	80.6	84.1	80.7	84.2	81.9	85.4
VR-2332	Lineage 5.1	AY150564.1	81.1	84.7	81.1	84.8	82.4	85.2
S1	Lineage 5.1	DQ459471	81.2	83.9	81.3	84.1	82.5	84.9
XW019	Lineage 6	KF724407.1	79.8	83.1	79.8	82.9	80.6	80.9
104194	Lineage 6	KT257957.1	76.6	83.1	76.6	82.6	77.2	82.4
SP	Lineage 7	AF184212	80.3	84.4	80.4	84.4	81.5	85.7
Prime_Pac	Lineage 7	DQ779791.1	80.3	84.4	80.3	84.4	81.5	85.7
APRRS	Lineage 7	GQ330474	80.2	84.6	80.3	84.6	81.4	86.1
PRRSV-201704176-1-S16	Lineage 7	MN073128	80.1	84.6	80.1	84.6	81.3	86.1
41761R-S15-L001	Lineage 7	MN073129.1	80.3	84.6	80.4	84.6	81.5	86.1
BJ0706	Lineage 8.7	GQ351601	79.7	82.9	79.8	83.1	80.7	84.7
JXA1	Lineage 8.7	EF112445	80.2	83.3	80.3	83.4	81.1	85.2
HUN4	Lineage 8.7	EF635006	80.2	83.4	80.3	83.6	81	85.4
TJ	Lineage 8.7	EU860248	80.4	83.4	80.4	83.6	81.1	85.4
YN9	Lineage 8.7	GU232738	80.4	83.1	80.5	83.3	81.2	85.4
NJ-1106	Lineage 8.7	JX880029	77.9	83.4	77.9	83.6	78.8	85.4
JXA1_P80	Lineage 8.7	FJ548853	80	83.3	80.1	83.4	80.9	85.2
JXA1-P170	Lineage 8.7	JQ804986	80.3	83.3	80.4	83.4	81.2	85.2
GX1003	Lineage 8.7	JX912249	80.3	83.4	80.4	83.6	81.1	85.4
JXA1-P120	Lineage 8.7	KC422727	80.3	83.3	80.4	83.4	81.1	85.2
CH-1a	Lineage 8.7	AY032626	80.5	84.9	80.6	84.7	81.5	86.4
CH-1R	Lineage 8.7	EU807840	80.5	84.2	80.6	84.1	81.6	85.7
CH2002	Lineage 8.7	EU880438	80	84.1	80.1	83.9	81.1	85.6
MN30100	Lineage 9	EF536000.1	79.3	84.1	79.4	83.9	80.3	84.4
SD95-10_P83	Lineage 9	KU131565.1	80.8	85.4	80.9	84.9	82	86.7
1692-98	Lineage 9	KY348847.1	80.9	83.9	81	84.1	82.1	84.4

Supplementary Table S5. Whole-genome sequencing metrics of the three PRRSV isolates

	DATA(%)	Avg depth	Median	Coverage%	Cov 10x %	Cov 100x %
SCABTC-202503	100	1091.69	1070	100	99.52	99.32
SCABTC-202505	100	3440.83	3523	99.7	99.65	98.97
SCABTC-202506	100	4106.72	4140	100	99.69	99.65

Data: Proportion of aligned data assigned to this contig;

Avg depth: Average alignment depth for this contig;

Median depth: Median alignment depth for this contig;

Coverage%: Alignment coverage of this contig;

Cov 10X: Percentage of this contig covered at a depth greater than 10×;

Cov 100X: Percentage of this contig covered at a depth greater than 100×.

Supplementary Table S6 Nucleotide differences between the isolated strains and their most closely related reference strains

Nucleotide identity %	SCABTC-202503			SCABTC-202505			SCABTC-202506		
Region	VR-2332	NADC30	CHsx1401	VR-2332	NADC30	CHsx1401	VR-2332	NADC30	CHsx1401
Nsp1	82.3	89	86.5	82.6	89	86.6	85	92.9	91.6
Nsp2	74	86.9	86.5	74.2	87.2	86.8	76.2	90.4	90.3
Nsp3	84.4	92.1	91.3	84.4	92.2	91.4	85.1	94.5	92.9
Nsp4	84.3	92.3	91.2	84.5	91.8	90.7	85.5	95.3	93.1
Nsp5	86.7	92.2	90.4	87.3	92.4	90.6	89.4	95.3	93.5
Nsp6	87.5	93.8	95.8	87.5	93.8	95.8	93.8	95.8	85.4
Nsp7	82	89.8	88	82	89.7	87.9	84.9	94.7	92.9
Nsp8	86.7	88.9	87.4	86.7	88.9	87.4	88.9	95.6	94.1
ORF1a	80.2	89.6	87.6	80.4	89.7	87.7	82.4	93.6	91.5
ORF1b	85.9	92.8	91.1	85.8	92.8	91.1	87.2	95.3	93
ORF2	86	90.1	89.1	85.9	90.4	89.4	85.6	91.3	90.1
ORF3	88.2	86	86.1	88.2	86.3	86.4	82.7	92.9	91.5
ORF4	86.4	93.1	91.8	86.2	92.9	91.6	87.9	94.4	93.7
ORF5	84.7	91.2	89.2	84.8	91	89	85.2	91.5	90
ORF6	88	94.3	93.3	89	94.9	93.9	88	93.3	92.6
ORF7	88.4	93.5	91.4	88.2	93.3	91.1	91.4	95.2	91.9

Supplementary Table S7 Amino acid differences between the isolated strains and their most closely related reference strains

Protein identity %	SCABTC-202503			SCABTC-202505			SCABTC-202506		
Region	VR-2332	NADC30	CHsx1401	VR-2332	NADC30	CHsx1401	VR-2332	NADC30	CHsx1401
Nsp1	84.6	91.4	89.3	85.4	90.9	89.6	86.9	91.9	90.3
Nsp2	63.9	83.7	82.7	63.9	84.2	83.1	66.3	87.9	87.1
Nsp3	90.2	94.2	93.7	90.2	94.4	94	89.9	96.4	95.1
Nsp4	93.1	95.6	96.1	93.1	95.6	96.1	93.6	96.1	96.6
Nsp5	86.5	92.4	92.4	86.5	92.4	92.4	93.5	96.5	95.3
Nsp6	100	100	93.8	100	100	93.8	100	100	93.8
Nsp7	84.6	91.1	90.3	84.9	91.5	90.7	87.6	95.4	93.4
Nsp8	95.6	88.9	91.1	95.6	88.9	91.1	93.3	95.6	97.8
ORF1a	80.4	90	87.9	80.6	90.2	88.2	82.8	93.1	90.6
ORF1b	94.7	97.3	96.4	94.4	97	96.2	95.7	97.9	97
ORF2	86.7	88.7	89.5	87.1	89.1	89.8	85.9	89.5	86.7
ORF3	84.6	85	84.6	85.4	85.8	85.8	81.9	89.8	87.8
ORF4	86	93.8	94.9	85.4	93.3	94.4	86.5	94.9	94.9
ORF5	81.4	91	88.9	82.4	90.5	88.9	84.5	92	90.5
ORF6	91.4	96.6	95.4	92.5	96.6	95.4	94.3	96.6	96.6
ORF7	89.4	92.7	89.4	88.6	91.9	88.6	91.9	95.1	90.2

Supplementary Table S8. Sanger sequencing results for the complete GP5 and Nsp2 sequences of first-passage and passage-10 cell-culture virus stocks

Name	Sequence
SCABTC-202503-GP5 (first-passage)	ATGTTGGGGAAATGCTTGACCGCGGGTTATTGCTCGCAATTGCCTTTTTTGTGGTGTATCGTGCCGT<br>TCTGCTTTGTTGTGCTCGTCAACGCCGACAGCAGCAGCTCCCATTTACAGTTGATTTATAACCTGAC<br>GATATGTGAGCTGAATGGCACGGATTGGCTGGGTGATCATTTTAGTTGGGCAGTGGAGACTTTTGT<br>CATCTTTCCTGCGTTGACTCACATTGTCTCTTACGGTGCCCTTACTACCAGCCATTTTCTTGACACG<br>GTCGGCCTCGCCGCTGTGTCCACCGCCGGTTACTATCACGGGCGGTATGTGTTGAGTAGCATCTAC<br>GCTGTCTGTGCTCTGGCTGCGTTTGTTTGCTTTGCTTTTCGATTGGCAAAAAATTGCATGTCCTGGC<br>GCTACTCATGCACTAGATATACCAATTTTCTCCTGGACACTAAGGGCAGACTCTACCGCTGGCGGT<br>CACCTGTCATCATAGAAAAAGGGGGTAAAGTTGATGTTGGGGATCAATTAATCGACCTCAAAAGA<br>GTTGTGCTTGAAGGCTCAGCGGCAACCCCTGTAACCAAGATTTCAGCGGAACAATGGGGTCGTCC<br>ATAG
SCABTC-202503-GP5 (passage-10)	ATGTTGGGGAAATGCTTGACCGCGGGTTATTGCTCGCAATTGCCTTTTTTGTGGTGTATCGTGCCGT<br>TCTGCTTTGTTGTGCTCGTCAACGCCGACAGCAGCAGCTCCCATTTACAGTTGATTTATAACCTGAC<br>GATATGTGAGCTGAATGGCACGGATTGGCTGGGTGATCATTTTAGTTGGGCAGTGGAGACTTTTGT<br>CATCTTTCCTGCGTTGACTCACATTGTCTCTTACGGTGCCCTTACTACCAGCCATTTTCTTGACACG<br>GTCGGCCTCGCCGCTGTGTCCACCGCCGGTTACTATCACGGGCGGTATGTGTTGAGTAGCATCTAC<br>GCTGTCTGTGCTCTGGCTGCGTTTGTTTGCTTTGCTTTTCGATTGGCAAAAAATTGCATGTCCTGGC<br>GCTACTCATGCACTAGATATACCAATTTTCTCCTGGACACTAAGGGCAGACTCTACCGCTGGCGGT<br>CACCTGTCATCATAGAAAAAGGGGGTAAAGTTGATGTTGGGGATCAATTAATCGACCTCAAAAGA<br>GTTGTGCTTGAAGGCTCAGCGGCAACCCCTGTAACCAAGATTTCAGCGGAACAATGGGGTCGTCC<br>ATAG
SCABTC-202505-GP5 (first-passage)	ATGTTGGGGAAATGCTTGACCGCGGGTTATTGCTTGCAATTGCTTTTTTTGTGGTGTATCGTGCCAT<br>TCTGCTTTGTTGTGCTCGTCAACGCCGACAACAGCAGCTCCCATTTACAGTCGATTTATAACCTGA<br>CGATATGTGAGCTGAATGGCACGGATTGGCTGGGTGATCATTTTAGTTGGGCAGTGGAAACTTTTG<br>TCATCTTTCCTGCGTTGACTCACATTGTCTCTTACGGTGCCCTTACTACCAGCCATTTTCTTGACAC<br>GGTCGGCCTCGCCACTGTGTCCACCGCCGGTTATTATCACGAGCGGTATGTGTTGAGTAGCATCTA<br>CGCTGTCTGTGCTCTGGCTGCGTTGGTTTGCTTTGCTTTTCGATTGGCAAAAAATTGCATGTCCTGG<br>CGCTACTCATGCACTAGATATACCAATTTTCTCCTGGACACTAAGGGCAGACTCTACCGCTGGCGG<br>TCACCTGTCATCATAGAAAAAGGGGGTAAAGTTGATGTTGGGGATCAATTAATCGACCTCAAAAGA<br>GTTGTGCTTGATGGCTCAGCGGCAACCCCTGTAACCAAGATTTCAGCGGAACAATGGGGTCGTCC<br>ATAG
SCABTC-202505-GP5 (passage-10)	ATGTTGGGGAAATGCTTGACCGCGGGTTATTGCTTGCAATTGCTTTTTTTGTGGTGTATCGTGCCAT<br>TCTGCTTTGTTGTGCTCGTCAACGCCGACAACAGCAGCTCCCATTTACAGTCGATTTATAACCTGA<br>CGATATGTGAGCTGAATGGCACGGATTGGCTGGGTGATCATTTTAGTTGGGCAGTGGAAACTTTTG<br>TCATCTTTCCTGCGTTGACTCACATTGTCTCTTACGGTGCCCTTACTACCAGCCATTTTCTTGACAC<br>GGTCGGCCTCGCCACTGTGTCCACCGCCGGTTATTATCACGAGCGGTATGTGTTGAGTAGCATCTA<br>CGCTGTCTGTGCTCTGGCTGCGTTGGTTTGCTTTGCTTTTCGATTGGCAAAAAATTGCATGTCCTGG<br>CGCTACTCATGCACTAGATATACCAATTTTCTCCTGGACACTAAGGGCAGACTCTACCGCTGGCGG<br>TCACCTGTCATCATAGAAAAAGGGGGTAAAGTTGATGTTGGGGATCAATTAATCGACCTCAAAAGA<br>GTTGTGCTTGATGGCTCAGCGGCAACCCCTGTAACCAAGATTTCAGCGGAACAATGGGGTCGTCC<br>ATAG

SCABTC-202506-GP5 (first-passage)	ATGTTGGGGAAATGCTTGACCGCGGGTTATTGCTCGCAATTGCTTTTTTTGTGGTGTATCGTGCCGT<br>TCTGTTCTGTTGCGCTCGTCAACGCCAACAGCAGCAGCAGCTCCCATATACAGTTGATTTACAACC<br>TGACGATATGTGAGCTGAATGGCACAGATTGGCTGAATGAAAAATTCGACTGGGCAGTGGAGACT<br>TTCGTCATTTTTCCTGTGTTGACCCACATTGTCTCTTATGGCGCCCTCACCACCAGCCATTTTCTTG<br>ACACGGTTGGCTTGGTCACTGTGTCCACCGCCGGGTATTACCACGGGCGATATGTGTTGAGTACCA<br>TCTATGCTGTCTGTGCCCTAGCTGCATTGGTATGCTTTGCCATTAGGTTGGCGAAGAATTGTATGTC<br>CTGGCGCTACTCATGCACCAGATATACCAACTTTCTTCTGGACACTAAGGGCAGACTCTACCGCTG<br>GCGGTCACCCGTCATCATTGAGAAGAGGGGCATGGTTGATGTGGGGGGTCATTTAATCGACCTCAA<br>GAAAGTTGTGCTTGACGGTTCCGCGGCAACTCCTGTAACCAGGATTTCAGCTGAGCAATGGGGTC<br>GTCCATAG
SCABTC-202506-GP5 (passage-10)	ATGTTGGGGAAATGCTTGACCGCGGGTTATTGCTCGCAATTGCTTTTTTTGTGGTGTATCGTGCCGT<br>TCTGTTCTGTTGCGCTCGTCAACGCCAACAGCAGCAGCAGCTCCCATATACAGTTGATTTACAACC<br>TGACGATATGTGAGCTGAATGGCACAGATTGGCTGAATGAAAAATTCGACTGGGCAGTGGAGACT<br>TTCGTCATTTTTCCTGTGTTGACCCACATTGTCTCTTATGGCGCCCTCACCACCAGCCATTTTCTTG<br>ACACGGTTGGCTTGGTCACTGTGTCCACCGCCGGGTATTACCACGGGCGATATGTGTTGAGTACCA<br>TCTATGCTGTCTGTGCCCTAGCTGCATTGGTATGCTTTGCCATTAGGTTGGCGAAGAATTGTATGTC<br>CTGGCGCTACTCATGCACCAGATATACCAACTTTCTTCTGGACACTAAGGGCAGACTCTACCGCTG<br>GCGGTCACCCGTCATCATTGAGAAGAGGGGCATGGTTGATGTGGGGGGTCATTTAATCGACCTCAA<br>GAAAGTTGTGCTTGACGGTTCCGCGGCAACTCCTGTAACCAGGATTTCAGCTGAGCAATGGGGTC<br>GTCCATAG
SCABTC-202503-Nsp2 (first-passage)	ATAACACACTCTAAATTTGAAACCACCCTTCCTGAAAGAGCGAGGTCTACCGAAGACTGGGCGAC<br>TGATGAGGATCTTGCTAACGCTATCCAAATTCTTAGGCTCCCTGCGGCCCTGGACAGGAATGGCGC<br>TTGTGCAGGTGCCAAGTATGTTCTCAAGCTGCAAGGCGAACATTGGACTGTCTCAGTTACTCCTGG<br>AATGTCTCCCTCCTTGCTTCCTCTAGAATGTGTTCAGGGCTGCTGCGAGCATAAGGGTGGCTCCAG<br>CTCCCCAAGCACGGTTGAAATTTCTGGGTTTGACCCTGCTTGCCTTGAATGGTTGGCCACAGTGAT<br>GCACTTGCCAAGTAGTATCATACCGGCTGCCCTGGCCGAGATGTCCGGCAACATCGGTCACTCGAC<br>CCCTCCGGTTTCTGGCGTGTGGACTGTTTCACAATTCTTCGCTAGTCACAATGAAGGGGAGCACTC<br>TGATCAAGTGTGCTTAAAGAAAATTGTCAGTCTTTGTCAACTGCTTGAGAACTGCTGCTGTTCTCA<br>GGACAAAGCCAACCCGGTCACCCCGGAGGAGGTGAGAGAAAAAATTAACCAGTACCTCTGCGGT<br>GCGGCAGATCTCGATGAATGCTTGGCTAGGCTTGAGAAAGCTCGCCCACCTAGCGTGTTGGACGC<br>CTCCTTTGATTGGAATGTCGTGTTTCCTGGGGTTGAAGTGGCTGCTCGAGCAGCGGAATCACCC<br>CTCATCAACCAGTGTTGCGCTCCAGTCGCTGCCGTGGCTCAAAAGCCACCGTCGGGATTCCAGTC<br>TAAAAGAGCGGAGTCTGTCAAGAGCCTCCCAGAGACCAGACCCCTCCCAGCCCCACGCAGGAAG<br>ACCAGGTCCATGTGTGGCAGTTTGGTGTCATTAGGTGGCAACTTTCCTGACAGCCGGGGTGACTC<br>GGCCAGTAGCCCTTTTCATTCCTCAGTCCTACCTAAGCCAGTGACACGTTCGAATGAAACTGTGCC<br>TGTCCCCGCACCGCGCAGGACTGTGTCCCGATCGAAGCTATCACCGACAGTGACAACCCCCGTGC<br>CCGCGCCACGATGTGGGCTTCAGCGGATGGAAGGGGCGAACTCGGCAGTAAGGACTCTGACGTG<br>CCAGGACGAGCTTCTAGATTTGTCTGCATCTTCGCAGACTGAATACGAGGCCCTCCCTTTGCCATT<br>GTCGCGGACTGAAGACGCCTTGGCGGTGAGGAGACATGAAGCTGAGGAAGTCCTTAGCGAAACC<br>GCGGACATGCCGGACGACACCAGGGAGACACCCGTGTCGTCGAGCAGCTCCCTGTCAAGTGTAG<br>AGATCACGCGCCCAAAATACTCAGCTCAAGCCATCATTGACACAGGTGGGCCCTGTTGCGGGCAC<br>CTCCAAGAGGTCAAGGAGAAATACCTTAATGTGATGCGTGAGGCATGTGATGCGACCAAGCTTGA<br>TCACCCTGCCACGCAAGAATGGCTTTCCCGCATGTGGGATAGGGTGGATATGCTGACCTGGCGCAA<br>CACGTCCATCTTTCAAGCGCCTTTCACCTTGGCCAACAAGTTCGAGTCTCTACCGAAGATGATACT

	TGAAACACCACCGCCCTACCCTTGCGGGTTCGTGATGATGCCCCGCACACCCGCGCCTTCCGTAG<br>GTGCGGAAAGCGACCTCACCGTTCGTTCAGTCGCCACTGAAGACGTTCCGTGCATTCTCGGAAAA<br>GCACACGATGTCAACAAGACGCTTGGCCAAGCACCCCTAGCACCCCCCACAGATGGATCAGCAGA<br>TGGCCGACTCGCCGGGGAACTCCAGACACAAGCTTCTCCCGCAGACACAGGTTGTGTTGATTTGG<br>GTTCGGATTCTGGAGGATCGCCGGAGCTTGCTGGCTTGTCGTCTCAAAACGGCACAGATGCAGGT<br>GGAGGGGGACTGTTGCACACGGTCAAGGACAAGGCTGAAAACTACTTTGACCGGTTGAATCGGC<br>GGGTTTTTGGCATCGTTTCCCATCTCCCTGTCTTCTTTTCACGTCTTTTCAGGTCCGATGATCATTAT<br>TCTCCGGGTGATTGGGGCTTTGCAGCTTTTACTTTATTGTGTCTCTTTCTATGTTACAGTTACCCGGC<br>GTTCGGTGCTGCCCCCCTTTTGGGTGTATTTTCTGGGACTCCTCGGCGCGTCCGCATGGGGGTTTTT<br>GGCTGCTGGCTGGCTTTTGCTGTTGGTTTGTTCAAGCCTGTGTCCGACCCAGTCGGCACTGCTTGT<br>GAGTTTGACTCGCCAGAATGTAGAGACATCCTTCATTCTTTTGAGCTTCTGCAACCTTGGGAACCT<br>GTTCGCTGCCTTGTGGTGGGGCCTGTCGGACTCTGCCTTGCCGTTCTTGGTAGGCTACTGGGCGGG<br>GCACGCTACATCTGGTTGCTTCTGCTTAGGCTTGGCATCCTATCAGACTGTATCCTGGCTGGAGCCT<br>ATGTGCTTTCGCAGGGCAGGTGCAAAAAGTGCTGGGGATCTTGTATAAGAACCGCCCCCAGCGAG<br>GTCGCTTTTAACGTGTTTCCCTTCACACGCGCGACCAGGTCATCACTCATCGACCTGTGCAATCGA<br>TTTTGTGTACCAAAAGGTATGGACCCCATCTTCCTCGCCACTGGATGGCGCGGGTGCTGGACCGGC<br>CAAAGCCCCATTGAGCAACCAACCGAGAAACCCATCGCGTATGCCCAATTGGATGAAAAGAAAAT<br>TACGGCCAAGACTGTGGTTGCCCAACCTTATGATCCCAATCAGGCTGTAAAGTGCTTGCGTGTTTT<br>GCAAGCTGGTGGGGCGATGGTTGCGGAGACTGTCCCAAAAGTGATTAAGGTTCATGCCATTCCATT<br>TCGAGCCCCTTTTCTTCCCACTGGGGTGAGGGTGGACCCTGATTGTAGGATTGTGGTCGATCCTGA<br>CACTTTCACAGCGGCTCTCCGGTCCGGCTATTCTACCTCAAACCTCGTCCTTGGTGTTGGGGATTTT<br>GCCCAGATGAATGGATTGAAAATCAGGCAGATCTCCAAGCCCTCAGGAGGGGGTCCGCACCTCGT<br>AGCTGCCCTGCATGTTGCTTGCTCGATGGCCATGCACATGCTTGCCGGGATTTACATGA
SCABTC-202503-Nsp2 (passage-10)	ATAACACACTCTAAATTTGAAACCACCCTTCCTGAAAGAGCGAGGTCTACCGAAGACTGGGCGAC<br>TGATGAGGATCTTGCTAACGCTATCCAAATTCTTAGGCTCCCTGCGGCCCTGGACAGGAATGGCGC<br>TTGTGCAGGTGCCAAGTATGTTCTCAAGCTGCAAGGCGAACATTGGACTGTCTCAGTTACTCCTGG<br>AATGTCTCCCTCCTTGCTTCCTCTAGAATGTGTTCAGGGCTGCTGCGAGCATAAGGGTGGCTCCAG<br>CTCCCCAAGCACGGTTGAAATTTCTGGGTTTGACCCTGCTTGCCTTGAATGGTTGGCCACAGTGAT<br>GCACTTGCCAAGTAGTATCATACCGGCTGCCCTGGCCGAGATGTCCGGCAACATCGGTCACTCGAC<br>CCCTCCGGTTTCTGGCGTGTGGACTGTTTCACAATTCTTCGCTAGTCACAATGAAGGGGAGCACTC<br>TGATCAAGTGTGCTTAAAGAAAATTGTCAGTCTTTGTCAACTGCTTGAGAACTGCTGCTGTTCTCA<br>GGACAAAGCCAACCCGGTCACCCCGGAGGAGGTGAGAGAAAAAATTAACCAGTACCTCTGCGGT<br>GCGGCAGATCTCGATGAATGCTTGGCTAGGCTTGAGAAAGCTCGCCCACCTAGCGTGTTGGACGC<br>CTCCTTTGATTGGAATGTCGTGTTTCCTGGGGTTGAAGTGGCTGCTCGAGCAGCGGAATCACCC<br>CTCATCAACCAGTGTTGCGCTCCAGTCGCTGCCGTGGCTCAAAAGCCACCGTCGGGATTCCAGTC<br>TAAAAGAGCGGAGTCTGTCAAGAGCCTCCCAGAGACCAGACCCCTCCCAGCCCCACGCAGGAAG<br>ACCAGGTCCATGTGTGGCAGTTTGGTGTCATTAGGTGGCAACTTTCCTGACAGCCGGGGTGACTC<br>GGCCAGTAGCCCTTTTCATTCCTCAGTCCTACCTAAGCCAGTGACACGTTCGAATGAAACTGTGCC<br>TGTCCCCGCACCGCGCAGGACTGTGTCCCGATCGAAGCTATCACCGACAGTGACAACCCCCGTGC<br>CCGCGCCACGATGTGGGCTTCAGCGGATGGAAGGGGCGAACTCGGCAGTAAGGACTCTGACGTG<br>CCAGGACGAGCTTCTAGATTTGTCTGCATCTTCGCAGACTGAATACGAGGCCCTCCCTTTGCCATT<br>GTCGCGGACTGAAGACGCCTTGGCGGTGAGGAGACATGAAGCTGAGGAAGTCCTTAGCGAAACC<br>GCGGACATGCCGGACGACACCAGGGAGACACCCGTGTCGTCGAGCAGCTCCCTGTCAAGTGTAG<br>AGATCACGCGCCCAAAATACTCAGCTCAAGCCATCATTGACACAGGTGGGCCCTGTTGCGGGCAC

	CTCCAAGAGGTCAAGGAGAAATACCTTAATGTGATGCGTGAGGCATGTGATGCGACCAAGCTTGA TCACCCTGCCACGCAAGAATGGCTTTCCCGCATGTGGGATAGGGTGGATATGCTGACCTGGCGCAA CACGTCCATCTTTCAAGCGCCTTTCACCTTGGCCAACAAGTTCGAGTCTCTACCGAAGATGATACT TGAAACACCACCGCCCTACCCTTGCGGGTTCGTGATGATGCCCCGCACACCCGCGCCTTCCGTAG GTGCGGAAAGCGACCTCACCGTTCGTTCAGTCGCCACTGAAGACGTTCCGTGCATTCTCGGAAAA GCACACGATGTCAACAAGACGCTTGGCCAAGCACCCCTAGCACCCCCCACAGATGGATCAGCAGA TGGCCGACTCGCCGGGGAACTCCAGACACAAGCTTCTCCCGCAGACACAGGTTGTGTTGATTTGG GTTCGGATTCTGGAGGATCGCCGGAGCTTGCTGGCTTGTCGTCTCAAAACGGCACAGATGCAGGT GGAGGGGGACTGTTGCACACGGTCAAGGACAAGGCTGAAAACTACTTTGACCGGTTGAATCGGC GGGTTTTTGGCATCGTTTCCCATCTCCCTGTCTTCTTTTCACGTCTTTTCAGGTCCGATGATCATTAT TCTCCGGGTGATTGGGGCTTTGCAGCTTTTACTTTATTGTGTCTCTTTCTATGTTACAGTTACCCGGC GTTCGGTGCTGCCCCCCTTTTGGGTGTATTTTCTGGGACTCCTCGGCGCGTCCGCATGGGGGTTTTT GGCTGCTGGCTGGCTTTTGCTGTTGGTTTGTTCAAGCCTGTGTCCGACCCAGTCGGCACTGCTTGT GAGTTTGACTCGCCAGAATGTAGAGACATCCTTCATTCTTTTGAGCTTCTGCAACCTTGGGAACCT GTTCGCTGCCTTGTGGTGGGGCCTGTCGGACTCTGCCTTGCCGTTCTTGGTAGGCTACTGGGCGGG GCACGCTACATCTGGTTGCTTCTGCTTAGGCTTGGCATCCTATCAGACTGTATCCTGGCTGGAGCCT ATGTGCTTTCGCAGGGCAGGTGCAAAAAGTGCTGGGGATCTTGTATAAGAACCGCCCCCAGCGAG GTCGCTTTTAACGTGTTTCCCTTCACACGCGCGACCAGGTCATCACTCATCGACCTGTGCAATCGA TTTTGTGTACCAAAAGGTATGGACCCCATCTTCCTCGCCACTGGATGGCGCGGGTGCTGGACCGGC CAAAGCCCCATTGAGCAACCAACCGAGAAACCCATCGCGTATGCCCAATTGGATGAAAAGAAAAT TACGGCCAAGACTGTGGTTGCCCAACCTTATGATCCCAATCAGGCTGTAAAGTGCTTGCGTGTTTT GCAAGCTGGTGGGGCGATGGTTGCGGAGACTGTCCCAAAAGTGATTAAGGTTCATGCCATTCCATT TCGAGCCCCTTTTCTTCCCACTGGGGTGAGGGTGGACCCTGATTGTAGGATTGTGGTCGATCCTGA CACTTTCACAGCGGCTCTCCGGTCCGGCTATTCTACCTCAAACCTCGTCCTTGGTGTTGGGGATTTT GCCCAGATGAATGGATTGAAAATCAGGCAGATCTCCAAGCCCTCAGGAGGGGGTCCGCACCTCGT AGCTGCCCTGCATGTTGCTTGCTCGATGGCCATGCACATGCTTGCCGGGATTTACATGA
SCABTC-202505-Nsp2 (first-passage)	ATAACACACTCTAAATTTGAAACCACCCTTCCTGAAAGAGCGAGGTCTACTGAAGACTGGGCGAC TGATGAGGATCTTGCTAACGCTATCCAAATTCTTAGGCTCCCTGCGGCCCTGGACAGGAATGGCGC TTGTGCAGGTGCCAAGTATGTCCTCAAGCTGCAAGGCGAACATTGGACTGTCTCAATTACTCCTGG AATGTCTCCCTCCTTGCTTCCTCTAGAATGTGTTCAGGGCTGCTGCGAGCATAAGGGTGGCTCCAG CTCCCCAAGCACGGTTGAAATTTCTGGGTTTGACCCTGCTTGCCTTGAATGGTTGGCCAAAGTGAT GCACTTGCCAAGTAGTATCATACCGGCTGCCCTGGCCGAGATGTCCGGCAACATCGGTCACTCGAC CCCTCCGGTTTCTGGCGTGTGGACTGTTTCACAATTCTTCGCTAGTCACAATGAAGGGGAGCACTC TGATCAAGTGTGCTTAAAGAAAATTGTCAGTCTTTGTCAACTGCTTGAGAACTGCTGCTGTTCTCA GGACAAAGCCAACCCGGTCACCCCGGAGGAGGTGAGAGAAAAAATTAACCAGTACCTCTGCGGT GCGGCAGATCTCGATGAATGCTTGGCTAGGCTTGAGAAAGCTCGCCCGCCTAGCGTGTTGGACGC CTCCTTTGATTGGAATGTCGTGTTTCCTGGGGTTGAAATGGCTGCTCGAGCAGCGGAATCACCCCT CATCAACCAGTGTCGCGCTCCAGTCGCTGCCGTGGCTCAAAAGCCACCGCCGGGATTCCAGTCTA GAAGAGCGGAGTCTGTCAAGAGCCTCCCAGAGACCAGACCCCTCCCTGCCCCACGCAGGAAGAC CAGGTCCATGTGTGGCAGTTTGGTGTCATTAGGTGGCAACTTTCCTGACAGCCGGGGTGACTCGG CCAGTGGCCCTTTTCATTCCTCAGTCCTACCTAAGCCGGTGACACGTTCGAATGAAACTGTGCCTG TCCCCGCACCGCGCAGGACTGTGTCCCGATCGAAGCTATCACCGACAGTGACAACCCCCGTGCCC GCGCCACGATGTGGGCTTCAGCGGATGGAAGGGGCGAACTCGGCAGTAGGGACTCTGACGTGCC AGGACGAGCTTCTAGATTTGTCTGCATCTTCGCAGACTGAATACGAGGCCCTCCCTTTGCCATTGT

	CGCGGACTGAAGACGCCCCGGCGGTGAGGAGACATGAAGCTGAGGAAGTCCTTAGCGAAACCGC<br>GGACATGCCGGACGACACCAGGGAGACACCCGTGTCGTCGAGCAGCTCCCTGTCAAGTGTAGAG<br>ATCACGCGCCCAAAATACTCAGCTCAAGCCATCATTGACACAGGTGGGCCCTGTTGCGGGCACCT<br>CCAAGAGGTCAAGGAGAAATACCTCAATGTGATGCGTGAGGCATGTGATGCGACCAAGCTTGATC<br>ACCCTGCCACGCAAGAATGGCTTTCCCGCATGTGGGATAGGGTGGATATGCTGACCTGGCGCAAC<br>ACGTCCATCTTTCAAGCGCCTTTCACCTTGGCCAACAAGTTCGAGTCTCTACCGAAGATGATACTT<br>GAAACACCACCGCCCTACCCTTGCGGGTTCGTGATGATGCCCCGCACACCCGCGCCTTCCGTAGG<br>TGCGGAAAGTGACCTCACCGTTTGTTCAGTCGCCACTGAAGACGTTCCGCGCATTCTCGGAAAAG<br>CACACGATGTCAACAAGACGCTTGACCAAGCACCCCTAGCACCCCCCACAGATGGATCAGCAGAT<br>GGCCGACTTGCTGGGGAACTCCAGACACAAGCTTCTCCCGCGGACATAGGTTGTGTTGATTTGGG<br>TTCGGATTCTGGAGGATCGCCGGAGCTTGCTGGCTTGTCGTCTCAAAACGGCACAGATGCAGGTG<br>GAGGGGGACTGTTGCACACGGTCAAGGACAAGGCTGAAAACTACTTTGACCGGTTGAATCGGCG<br>GGTTTTTGGCATCGTTTCCCATCTCCCTGTCTTCTTTTCACGTCTTTTCAGGTCCGATGATCATTATT<br>CTCCGGGTGATTGGGGCTTTGCAGCTTTTACTTTATTGTGTCTCTTTCTATGTTACAGTTACCCGGCG<br>TTCGGTGCTGCTCCCCTTGTGGGTGTATTTTCTGGGACTCCTCGGCGCGTCCGCATGGGGGTTTTT<br>GGCTGCTGGCTGGCTTTTGCTGTTGGTTTGTTCAAGCCTGTGTCCGACCCAGTCGGCACTGCTTGT<br>GAGTTTGACTCGCCAGAATGTAGAGACATCCTTCATTCTTTTGAGCTTCTGCAACCTTGGGAACCT<br>GTTCGCTGCCTTGTGGTGGGGCCTGTCGGACTCTGCCTTGCCGTCCTTGGTAGGCTACTGGGCGGG<br>GCACGCTACATCTGGTTGCTTCTGCTTAGGCTTGGCATCCTATCAGACTGTATTCTGGCTGGAGCCT<br>ATGTGCTTTCGCAGGGCAGGTGCAAAAAGTGCTGGGGATCTTGTATAAGAACCGCCCCCAGCGAG<br>GTCGCTTTTAACGTGTTTCCCTTCACACGCGCGACCAGGTCATCACTCATCGACCTGTGCAATCGA<br>TTTTGTGTACCAAAAGGTATGGACCCCATCTTCCTCGCCACTGGATGGCGCGGGTGCTGGACCGGC<br>CAAAGCCCCATTGAGCAACCAACTGAGAAACCCATCGCGTATGCCCAATTGGATGAAAAGAAAAT<br>TACGGCCAAGACTGTGGTTGCCCAACCTTATGATCCCAATCAGGCTGTAAAGTGCTTGCGTGTTTT<br>GCAAGCTGGTGGGGCGATGGTTGCGGAGACTGTCCCAAAAGTGACTAAGGTTCATGCCATTCCAT<br>TTCGAGCCCCTTTTCTTCCCACTGGGGTGAGGGTGGACCCTGATTGTAGGATTGTGGTCGATCCT<br>GACACTTTCACAGCGGCTCTCCGGTCCGGCTATTCTACCTCAAACCTCGTCCTTGGTGTTGGGGAT<br>TTTGCCCAGATGAATGGATTGAAAATCAGGCAGATCTCCAAGCCCTCAGGAGGGGGTCCGCACCT<br>CGTAGCTGCCCTGCATGTTGCTTGCTCGATGGCCATGCACATGCTTGCCGGGATTTACATGA
SCABTC-<br>202505-Nsp2<br>(passage-10)	ATAACACACTCTAAATTTGAAACCACCCTTCCTGAAAGAGCGAGGTCTACTGAAGACTGGGCGAC<br>TGATGAGGATCTTGCTAACGCTATCCAAATTCTTAGGCTCCCTGCGGCCCTGGACAGGAATGGCGC<br>TTGTGCAGGTGCCAAGTATGTCCTCAAGCTGCAAGGCGAACATTGGACTGTCTCAATTACTCCTGG<br>AATGTCTCCCTCCTTGCTTCCTCTAGAATGTGTTCAGGGCTGCTGCGAGCATAAGGGTGGCTCCAG<br>CTCCCCAAGCACGGTTGAAATTTCTGGGTTTGACCCTGCTTGCCTTGAATGGTTGGCCAAAGTGAT<br>GCACTTGCCAAGTAGTATCATACCGGCTGCCCTGGCCGAGATGTCCGGCAACATCGGTCACTCGAC<br>CCCTCCGGTTTCTGGCGTGTGGACTGTTTCACAATTCTTCGCTAGTCACAATGAAGGGGAGCACTC<br>TGATCAAGTGTGCTTAAAGAAAATTGTCAGTCTTTGTCAACTGCTTGAGAACTGCTGCTGTTCTCA<br>GGACAAAGCCAACCCGGTCACCCCGGAGGAGGTGAGAGAAAAAATTAACCAGTACCTCTGCGGT<br>GCGGCAGATCTCGATGAATGCTTGGCTAGGCTTGAGAAAGCTCGCCCGCCTAGCGTGTTGGACGC<br>CTCCTTTGATTGGAATGTCGTGTTTCCTGGGGTTGAAATGGCTGCTCGAGCAGCGGAATCACCCCT<br>CATCAACCAGTGTCGCGCTCCAGTCGCTGCCGTGGCTCAAAAGCCACCGCCGGGATTCCAGTCTA<br>GAAGAGCGGAGTCTGTCAAGAGCCTCCCAGAGACCAGACCCCTCCCTGCCCCACGCAGGAAGAC<br>CAGGTCCATGTGTGGCAGTTTGGTGTCATTAGGTGGCAACTTTCCTGACAGCCGGGGTGACTCGG<br>CCAGTGGCCCTTTTCATTCCTCAGTCCTACCTAAGCCGGTGACACGTTCGAATGAAACTGTGCCTG

	TCCCCGCACCGCGCAGGACTGTGTCCCGATCGAAGCTATCACCGACAGTGACAACCCCCGTGCCC<br>GCGCCACGATGTGGGCTTCAGCGGATGGAAGGGGCGAACTCGGCAGTAGGGACTCTGACGTGCC<br>AGGACGAGCTTCTAGATTTGTCTGCATCTTCGCAGACTGAATACGAGGCCCTCCCTTTGCCATTGT<br>CGCGGACTGAAGACGCCCCGGCGGTGAGGAGACATGAAGCTGAGGAAGTCCTTAGCGAAACCGC<br>GGACATGCCGGACGACACCAGGGAGACACCCGTGTCGTCGAGCAGCTCCCTGTCAAGTGTAGAG<br>ATCACGCGCCCAAAATACTCAGCTCAAGCCATCATTGACACAGGTGGGCCCTGTTGCGGGCACCT<br>CCAAGAGGTCAAGGAGAAATACCTCAATGTGATGCGTGAGGCATGTGATGCGACCAAGCTTGATC<br>ACCCTGCCACGCAAGAATGGCTTTCCCGCATGTGGGATAGGGTGGATATGCTGACCTGGCGCAAC<br>ACGTCCATCTTTCAAGCGCCTTTCACCTTGGCCAACAAGTTCGAGTCTCTACCGAAGATGATACTT<br>GAAACACCACCGCCCTACCCTTGCGGGTTCGTGATGATGCCCCGCACACCCGCGCCTTCCGTAGG<br>TGCGGAAAGTGACCTCACCGTTTGTTCAGTCGCCACTGAAGACGTTCCGCGCATTCTCGGAAAAG<br>CACACGATGTCAACAAGACGCTTGACCAAGCACCCCTAGCACCCCCCACAGATGGATCAGCAGAT<br>GGCCGACTTGCTGGGGAACTCCAGACACAAGCTTCTCCCGCGGACATAGGTTGTGTTGATTTGGG<br>TTCGGATTCTGGAGGATCGCCGGAGCTTGCTGGCTTGTCGTCTCAAAACGGCACAGATGCAGGTG<br>GAGGGGGACTGTTGCACACGGTCAAGGACAAGGCTGAAAACTACTTTGACCGGTTGAATCGGCG<br>GGTTTTTGGCATCGTTTCCCATCTCCCTGTCTTCTTTTCACGTCTTTTCAGGTCCGATGATCATTATT<br>CTCCGGGTGATTGGGGCTTTGCAGCTTTTACTTTATTGTGTCTCTTTCTATGTTACAGTTACCCGGCG<br>TTCGGTGCTGCTCCCCTTGTGGGTGTATTTTCTGGGACTCCTCGGCGCGTCCGCATGGGGGTTTTT<br>GGCTGCTGGCTGGCTTTTGCTGTTGGTTTGTTCAAGCCTGTGTCCGACCCAGTCGGCACTGCTTGT<br>GAGTTTGACTCGCCAGAATGTAGAGACATCCTTCATTCTTTTGAGCTTCTGCAACCTTGGGAACCT<br>GTTCGCTGCCTTGTGGTGGGGCCTGTCGGACTCTGCCTTGCCGTCCTTGGTAGGCTACTGGGCGGG<br>GCACGCTACATCTGGTTGCTTCTGCTTAGGCTTGGCATCCTATCAGACTGTATTCTGGCTGGAGCCT<br>ATGTGCTTTCGCAGGGCAGGTGCAAAAAGTGCTGGGGATCTTGTATAAGAACCGCCCCCAGCGAG<br>GTCGCTTTTAACGTGTTTCCCTTCACACGCGCGACCAGGTCATCACTCATCGACCTGTGCAATCGA<br>TTTTGTGTACCAAAAGGTATGGACCCCATCTTCCTCGCCACTGGATGGCGCGGGTGCTGGACCGGC<br>CAAAGCCCCATTGAGCAACCAACTGAGAAACCCATCGCGTATGCCCAATTGGATGAAAAGAAAAT<br>TACGGCCAAGACTGTGGTTGCCCAACCTTATGATCCCAATCAGGCTGTAAAGTGCTTGCGTGTTTT<br>GCAAGCTGGTGGGGCGATGGTTGCGGAGACTGTCCCAAAAGTGACTAAGGTTCATGCCATTCCAT<br>TTCGAGCCCCTTTTCTTCCCACTGGGGTGAGGGTGGACCCTGATTGTAGGATTGTGGTCGATCCT<br>GACACTTTCACAGCGGCTCTCCGGTCCGGCTATTCTACCTCAAACCTCGTCCTTGGTGTTGGGGAT<br>TTTGCCCAGATGAATGGATTGAAAATCAGGCAGATCTCCAAGCCCTCAGGAGGGGGTCCGCACCT<br>CGTAGCTGCCCTGCATGTTGCTTGCTCGATGGCCATGCACATGCTTGCCGGGATTTACATGA
SCABTC-<br>202506-Nsp2<br>(first-passage)	ATGGTACATTCTAAATTCGAAACCACCCTTCCTGAAAGAGTGAGGTCCCCAGATGACTGGGCGAC<br>TGATGAAGATCTTGCTAACGCTATCCAAACCCTCAGGCTCCCTGCGGCCTTGGACAGGAATGGTGC<br>TTGTGCAGGCGCCAAGTACGTTCTCAAGCTGGAAGGCGAGCATTGGACTGTCTCAGTTACCCCTG<br>GGATGTCTCCCTCTTTGCTTCCTCTTGAATGTGTTCAGGGTTGCTGTGAGCACAAGGGTGGCTCTA<br>GCCCCCCAAGCGCGGTTGAAATTTCTGGGTTTGACCCTGCCTGCCTTGATTGGTTGGCCAAAGTGA<br>TGCACCTGCCTAGCAGTGTTATCCCGGCCGCCTTGGCCAATATGTCCGGTAACATCGGTCATTTGAC<br>ACCTCCGGTTTCTGGTGTGTGGACTGTCTCACAACTCTTCGCTCGTCACAGCGGAGGGGAGCATT<br>CTGATCAAGTGTACTTGGAGAAAATTGTCAGCCTTTGTCAACTGCTTGAGAGTTGCTGCTGTTCTC<br>AGAACAAAGCCAACCCGGTCACCCCGGAGGAGGTGAGGGAAAAGATTGACCAGTACCTCCGCGG<br>TGCGGTAAATCTTGAAGAATGCTTGGCTAGGCTTGAGAAAGCTCGCCCGCCTAGCGTGTTGGACA<br>CCTCCTTTGATTGGGATGTCGTGTTTCCTGGAGTCGGAGTGGCTGCTAAAGCAGCGGAACTGCCCC<br>TCATCAACCAGTGCCACGCTCCAGTCACTGTCGTGGCCCGAAGGTCTTCGCCGGAACTCCAGTCT

	CGAAAAGCGGAGTCTGTCAAGAGCCTCCCAGAGAACAGGCCCCTCCCTGCCCCACGCAGGAAGA<br>TCAGGTCCATGTGTGGTAGTTTGGTTTCATCAGGTGACAACCACCCTGACAGCCGGGAAGACTCG<br>GCCGGTGCCTCCTTTCATTCTCCAGTCCCGTCTAAGTCAGTGGCACGTTCGAATGAACCTGCGCCT<br>GTCCCCGCACCGCGCAGGACTGTGTCCCGACTGAAGCTATCACCGACAATGACAACCCCTGCGCC<br>CGCACCACGATGTGAGCTTCAGCGGGTGGAAGGGGTGAACTCAGCAGTAGGGACTCTGGCGTAC<br>CAGGACGAGCTCCTCGATTTGTCTGCATCCTCGCTGACTGCATATGAGACTTCCCCTTTGGCATTGC<br>CGCAGAGTGAGGACGCCCTGGCGGTGAGGAGACGTGAAGCTGAGGAAGTCCCTAGCGAAGCCA<br>CGGATATGTCGGACGACATCAGGTTGGCACCCGTGTCATCAAGCAGTTCCCTGTCAAGCGTAGAG<br>ATCACGCGCCCAAAATATTCAGCTCAAGCCATCATTGACGCACGTGGACCCTGTTGTGGGCACCTC<br>CAAGAGGTAAAGGAGAAATACCTCAATGTGATGCGTGAGGCATGTGATGCGACCAAGCTTGATGA<br>CCCTGCCACGCAAGAATGGCTTTCCCGCATGTGGGATAGGGTGGACATGCTAACCTGGCGCAACA<br>CGTCCATCTTTCAAGCGCCTTTCACCTTGGCCAACAAGTTCGAGTCTCTACCGAAGATGATACTTG<br>AAACACCACCGCCCTACCCTTGCGGGTTTGTGATGATGCCCCGCACACCAGTGCCTTCCGTAGGTG<br>CGGAGAGCGACCTCACCGTTGGTTCAGTCGCCACTGAAGACGTTCCGCGCATTCTCGGAAAGGTA<br>CACGATGTTAGCAAAACGGCCGACCAGGCACCCTTAGCACCCTCCACAGATGGATCAACAGATGG<br>CCGACTTGCTGGGGAACCCCGGACACAAGATCCTCCCGTAGGCACAGGTGGCGTTGACTTAGGCT<br>CGGATTCTGGAGGATTGCCGGAGCTCGCTGGCTTGTCGTCTAAAAACGGTACAGATGCAGGTGGA<br>GGGGGACTGTTACACACGGTCAAGGATAAGGCTGAGAAATGCTTTGACCAGCTGAGTCGGCGGG<br>TTTTTGGCATCGTCTCCCATCTCCCTGTCTTCTTCTCACGCTTGTTCAGGTCCGACGGTTATTATTCT<br>CCGGGTGATTGGGGCTTTGCAACTTTTACTTTATTGTGTCTCTTTCTATGTTACAGTTACCCTGCGTT<br>TGGTGTCGCTCCCCTCGTGGGTGTATTTTCTGGGACTCCTCGGCGCGTCCGCATGGGGGTTTTTGG<br>CTGCTGGCTGGCTTTTGCTGTTGGTTTGTTCAAGCCTGTACCCGACCCAGTCGGCACTGCTTGTGA<br>ATTTGACTCGCCAGAATGTAGAGACATCCTTCATTCTTTTGAGCTTCTGCAACCTTGGGAACCTGTT<br>CGCAGCCTTGTGGTGGGGCCCGTCGGGCTCTGCCTCGCAGTTCTTGGTAGGTTACTGGGTGGGGC<br>ACGCCACGTCTGGTTGCTTCTGCTTAGGCTTGGCATCCTATCAGACTGTGTTCTGGCTGGAGCCTAT<br>GTGCTTTCGCAGGGCAGGTGCAAAAAGTGCTGGGGATCTTGTATAAGAACTGCCCCCAGCGAGGT<br>CGCTTTTAACGTGTTTCCCTTCACACGTGCGACCAGGTCATCACTCATCGATTTGTGCAATCGATTT<br>TGTGTACCAAAGGGCATGGACCCCATTCTCCTCGCCACTGGATGGCGCGGGTGCTGGACCGGACA<br>AAGCCCCATTGAGCAGCCATCTGAGAAACCCATCGCGTATGCCCAATTGGATGAAAAGAAAATTA<br>CGGCTAGGACTGTGGTCGCCCAACCTTATGATCCCAATCAGGCTGTAAAGTGCTTACGGGTTTTGC<br>AGGCTGGTGGGGTGATGGTGGCGGAGGCTGTCCCAAAAGTGACTAAGGTTTCTGCTATTCCATTTC<br>GAGCCCCTTTTTTTCCCACTGGGGTGAGAGTTGACCCCGATTGCAGGATTGTGGTCGATCCTGACA<br>CTTTTACAGCGGCTCTCCGGTCCGGCTATTCTACTTCAAACCTCATCCTTGGTGTTGGGGATTTTGC<br>CCAGCTGAATGGATTGAAAATCAGGCAGATCTCCAAGTCCTCAGGAGGAGGCCCGCACTTCGCAG<br>CTGCCCTGCATGTCGCTTGCTCGATGGCCATGCACCTGCTTGCCGGGATTTATGTAA
SCABTC-202506-Nsp2 (passage-10)	ATGGTACATTCTAAATTCGAAACCACCCTTCCTGAAAGAGTGAGGTCCCCAGATGACTGGGCGAC<br>TGATGAAGATCTTGCTAACGCTATCCAAACCCTCAGGCTCCCTGCGGCCTTGGACAGGAATGGTGC<br>TTGTGCAGGCGCCAAGTACGTTCTCAAGCTGGAAGGCGAGCATTGGACTGTCTCAGTTACCCCTG<br>GGATGTCTCCCTCTTTGCTTCCTCTTGAATGTGTTCAGGGTTGCTGTGAGCACAAGGGTGGCTCTA<br>GCCCCCCAAGCGCGGTTGAAATTTCTGGGTTTGACCCTGCCTGCCTTGATTGGTTGGCCAAAGTGA<br>TGCACCTGCCTAGCAGTGTTATCCCGGCCGCCTTGGCCAATATGTCCGGTAACATCGGTCATTTGAC<br>ACCTCCGGTTTCTGGTGTGTGGACTGTCTCACAACTCTTCGCTCGTCACAGCGGAGGGGAGCATT<br>CTGATCAAGTGTACTTGGAGAAAATTGTCAGCCTTTGTCAACTGCTTGAGAGTTGCTGCTGTTCTC<br>AGAACAAAGCCAACCCGGTCACCCCGGAGGAGGTGAGGGAAAAGATTGACCAGTACCTCCGCGG

	TGCGGTAAATCTTGAAGAATGCTTGGCTAGGCTTGAGAAAGCTCGCCCGCCTAGCGTGTTGGACA<br>CCTCCTTTGATTGGGATGTCGTGTTTCCTGGAGTCGGAGTGGCTGCTAAAGCAGCGGAACTGCCCC<br>TCATCAACCAGTGCCACGCTCCAGTCACTGTCGTGGCCCGAAGGTCTTCGCCGGAACTCCAGTCT<br>CGAAAAGCGGAGTCTGTCAAGAGCCTCCCAGAGAACAGGCCCCTCCCTGCCCCACGCAGGAAGA<br>TCAGGTCCATGTGTGGTAGTTTGGTTTCATCAGGTGACAACCACCCTGACAGCCGGGAAGACTCG<br>GCCGGTGCCTCCTTTCATTCTCCAGTCCCGTCTAAGTCAGTGGCACGTTCGAATGAACCTGCGCCT<br>GTCCCCGCACCGCGCAGGACTGTGTCCCGACTGAAGCTATCACCGACAATGACAACCCCTGCGCC<br>CGCACCACGATGTGAGCTTCAGCGGGTGGAAGGGGTGAACTCAGCAGTAGGGACTCTGGCGTAC<br>CAGGACGAGCTCCTCGATTTGTCTGCATCCTCGCTGACTGCATATGAGACTTCCCCTTTGGCATTGC<br>CGCAGAGTGAGGACGCCCTGGCGGTGAGGAGACGTGAAGCTGAGGAAGTCCCTAGCGAAGCCA<br>CGGATATGTCGGACGACATCAGGTTGGCACCCGTGTCATCAAGCAGTTCCCTGTCAAGCGTAGAG<br>ATCACGCGCCCAAAATATTCAGCTCAAGCCATCATTGACGCACGTGGACCCTGTTGTGGGCACCTC<br>CAAGAGGTAAAGGAGAAATACCTCAATGTGATGCGTGAGGCATGTGATGCGACCAAGCTTGATGA<br>CCCTGCCACGCAAGAATGGCTTTCCCGCATGTGGGATAGGGTGGACATGCTAACCTGGCGCAACA<br>CGTCCATCTTTCAAGCGCCTTTCACCTTGGCCAACAAGTTCGAGTCTCTACCGAAGATGATACTTG<br>AAACACCACCGCCCTACCCTTGCGGGTTTGTGATGATGCCCCGCACACCAGTGCCTTCCGTAGGTG<br>CGGAGAGCGACCTCACCGTTGGTTCAGTCGCCACTGAAGACGTTCCGCGCATTCTCGGAAAGGTA<br>CACGATGTTAGCAAAACGGCCGACCAGGCACCCTTAGCACCCTCCACAGATGGATCAACAGATGG<br>CCGACTTGCTGGGGAACCCCGGACACAAGATCCTCCCGTAGGCACAGGTGGCGTTGACTTAGGCT<br>CGGATTCTGGAGGATTGCCGGAGCTCGCTGGCTTGTCGTCTAAAAACGGTACAGATGCAGGTGGA<br>GGGGGACTGTTACACACGGTCAAGGATAAGGCTGAGAAATGCTTTGACCAGCTGAGTCGGCGGG<br>TTTTTGGCATCGTCTCCCATCTCCCTGTCTTCTTCTCACGCTTGTTCAGGTCCGACGGTTATTATTCT<br>CCGGGTGATTGGGGCTTTGCAACTTTTACTTTATTGTGTCTCTTTCTATGTTACAGTTACCCTGCGTT<br>TGGTGTCGCTCCCCTCGTGGGTGTATTTTCTGGGACTCCTCGGCGCGTCCGCATGGGGGTTTTTGG<br>CTGCTGGCTGGCTTTTGCTGTTGGTTTGTTCAAGCCTGTACCCGACCCAGTCGGCACTGCTTGTGA<br>ATTTGACTCGCCAGAATGTAGAGACATCCTTCATTCTTTTGAGCTTCTGCAACCTTGGGAACCTGTT<br>CGCAGCCTTGTGGTGGGGCCCGTCGGGCTCTGCCTCGCAGTTCTTGGTAGGTTACTGGGTGGGGC<br>ACGCCACGTCTGGTTGCTTCTGCTTAGGCTTGGCATCCTATCAGACTGTGTTCTGGCTGGAGCCTAT<br>GTGCTTTCGCAGGGCAGGTGCAAAAAGTGCTGGGGATCTTGTATAAGAACTGCCCCCAGCGAGGT<br>CGCTTTTAACGTGTTTCCCTTCACACGTGCGACCAGGTCATCACTCATCGATTTGTGCAATCGATTT<br>TGTGTACCAAAGGGCATGGACCCCATTCTCCTCGCCACTGGATGGCGCGGGTGCTGGACCGGACA<br>AAGCCCCATTGAGCAGCCATCTGAGAAACCCATCGCGTATGCCCAATTGGATGAAAAGAAAATTA<br>CGGCTAGGACTGTGGTCGCCCAACCTTATGATCCCAATCAGGCTGTAAAGTGCTTACGGGTTTTGC<br>AGGCTGGTGGGGTGATGGTGGCGGAGGCTGTCCCAAAAGTGACTAAGGTTTCTGCTATTCCATTTC<br>GAGCCCCTTTTTTTCCCACTGGGGTGAGAGTTGACCCCGATTGCAGGATTGTGGTCGATCCTGACA<br>CTTTTACAGCGGCTCTCCGGTCCGGCTATTCTACTTCAAACCTCATCCTTGGTGTTGGGGATTTTGC<br>CCAGCTGAATGGATTGAAAATCAGGCAGATCTCCAAGTCCTCAGGAGGAGGCCCGCACTTCGCAG<br>CTGCCCTGCATGTCGCTTGCTCGATGGCCATGCACCTGCTTGCCGGGATTTATGTAA

Supplementary Table S9. RDP4-based recombination analysis of the isolated PRRSV strains

Recombinant strain	parental strain	Recombinant breakpoint (nt)	The p-value of the recombination analysis method						
			RDP	GENECONV	BootScan	MaxChi	Chimaera	SiScan	3seq
SCABTC-202503	NADC30/ VR2332	12705-13156	5.66×10^{-23}	4.62×10^{-08}	1.642×10^{-21}	9.211×10^{-9}	1.258×10^{-10}	1.485×10^{-7}	ns
SCABTC-202505	NADC30/ VR2332	12705-13156	2.269×10^{-23}	ns	6.691×10^{-22}	3.809×10^{-9}	5.628×10^{-11}	1.116×10^{-7}	ns
SCABTC-202506	NADC30/ VR2332	7866-8252	9.697×10^{-9}	ns	9.663×10^{-9}	5.418×10^{-6}	6.025×10^{-7}	2.280×10^{-2}	ns
